# Fast quantum interference of a nanoparticle via optical potential control

**DOI:** 10.1073/pnas.2306953121

**Published:** 2024-01-16

**Authors:** Lukas Neumeier, Mario A. Ciampini, Oriol Romero-Isart, Markus Aspelmeyer, Nikolai Kiesel

**Affiliations:** ^a^Vienna Center for Quantum Science and Technology, Faculty of Physics, University of Vienna, Vienna A-1090, Austria; ^b^Institute for Quantum Optics and Quantum Information (IQOQI) Innsbruck, Austrian Academy of Sciences, Innsbruck A-6020, Austria; ^c^Institute for Theoretical Physics, School of Mathematics, Computer Science and Physics, University of Innsbruck, Innsbruck A-6020, Austria; ^d^Institute for Quantum Optics and Quantum Information (IQOQI) Vienna, Austrian Academy of Sciences, Vienna A-1090, Austria

**Keywords:** optomechanics, matter–wave, nonlinear dynamics

## Abstract

We introduce and theoretically analyze a scheme to prepare and detect non-Gaussian quantum states of an optically levitated particle via the interaction with light pulses that generate cubic and inverted potentials. We show that this approach allows to operate on sufficiently short time- and length scales to beat decoherence in a regime accessible in state-of-the-art experiments. Specifically, we predict the observation of single-particle interference of a nanoparticle with a mass above 10^8^ atomic mass units delocalized by several nanometers, on timescales of milliseconds. The proposed experiment uses only optical and electrostatic control, and can be performed at about 10^−10^ mbar and at room temperature. We discuss the prospect of this method for coherently splitting the wavepacket of massive dielectric objects without using either projective measurements or an internal level structure.

## Significance

Testing the quantum superposition principle for large delocalization and increasingly massive objects is one of the grand challenges of modern quantum experiments. Levitated solid-state particles have recently emerged as a promising system to achieve this. In this work, we introduce an experimental method to generate center-of-mass superposition states at previously unattainable mass-, length- and time scales using only external optical and electrostatic potentials. We analyze relevant decoherence mechanisms and show the feasibility of observing quantum interference of a largely delocalized 100-nm silica particle at room temperature and with available state-of-the-art technology. Our results provide a route to experimentally realizing quantum superpositions of large mass and macroscopic separation.

Exploring quantum physics at increasingly macroscopic scales is fundamentally motivated by the curiosity about how far we can push counterintuitive quantum phenomena into the realm of our everyday experience (1–3). In addition, quantum superposition states involving large mass and macroscopic separation allow for highly sensitive probes of ultra-weak forces, e.g., as sensors for new physics beyond the standard model (4–8) or even for nonclassical sources of gravity to investigate the gravity–quantum interface (9, 10). Generating such states in the laboratory, however, remains an outstanding challenge. Experiments involving superposition states of Bose-Einstein Condensates (BECs) (11), macromolecules (12), and even solid state systems (13–16) constitute important milestones in this direction, yet in all these cases, realizing large mass seems to prevent large separation in the superposition, and vice versa. A promising platform to overcome this limitation is motional quantum control of levitated solids in high vacuum (17, 18). Only recently, different cooling methods enabled the preparation of the quantum ground state of motion of dielectric nanoparticles in the mass range of 10^8^ atomic mass units (a.m.u.) (19–22). In parallel, spatiotemporal control of optical traps has been developed. It allows for the rapid manipulation of complex potential landscapes (23), expanding the available toolbox of optical levitation into the regime of nonlinear dynamics (24). Here, we combine these developments into an experimental protocol to generate non-Gaussian center-of-mass superposition states at previously unattainable mass and time scales, hence offering access to the high-mass regime of quantum superpositions at macroscopic separation only by controlling optical potentials.

It is well known that dynamics in nonharmonic potentials allow for quantum state preparation beyond the Gaussian realm, providing access to genuine quantum features like negative quasi-probabilities (11, 25–28). One may be skeptical, however, if the nonlinear features of diffraction-limited optical potentials can be exploited to generate non-Gaussian quantum states given that nanoparticle wavepackets are notoriously small (compared to the optical wavelength) and subject to decoherence from light scattering. In this work, we argue that the answer is positive. We overcome both possible showstoppers by using only short interaction times with nonlinear optical potentials and by letting the system freely evolve in-between, which minimizes decoherence by scattering and increases the wavepacket size. In essence, the pulsed light–matter interaction creates a phase modulation of the particle wavepacket (29). For a cubic potential (and potentials of higher order), this is equivalent to interacting with a sub-wavelength diffraction grating that prepares the desired delocalized non-Gaussian quantum state. This can be probed in a subsequent measurement through observing a spatial interference pattern. We predict specifically that for a silica particle of radius r=50 nm (mass of ∼ 6 · 10^8^ a.m.u.), interference is observable within a few milliseconds. Since operating on such a short time scale dramatically reduces the requirements on decoherence due to gas pressure and blackbody radiation (30), our scheme is predicted to work in a room temperature environment and ultra-high vacuum level (10^−10^ mbar). In comparison to other approaches, our scheme neither relies on external nonlinearities, e.g., by coupling to external qubits (31–33) or induced by measurement (34–36), nor on internal degrees of freedom (37–42). It only exploits the evolution in a fast, time-varying potential landscape. This also makes it fundamentally different from Talbot–Lau interferometry (43, 44), where the Talbot time limits the accessible mass range to well below 10^8^ a.m.u. Our proposal is the first deterministic scheme based only on external potentials to realize mass superpositions in a mass range beyond 10^8^ a.m.u. and at time scales fast enough to manage decoherence effects at moderate pressures and at room temperature.

**Fig. 1.**
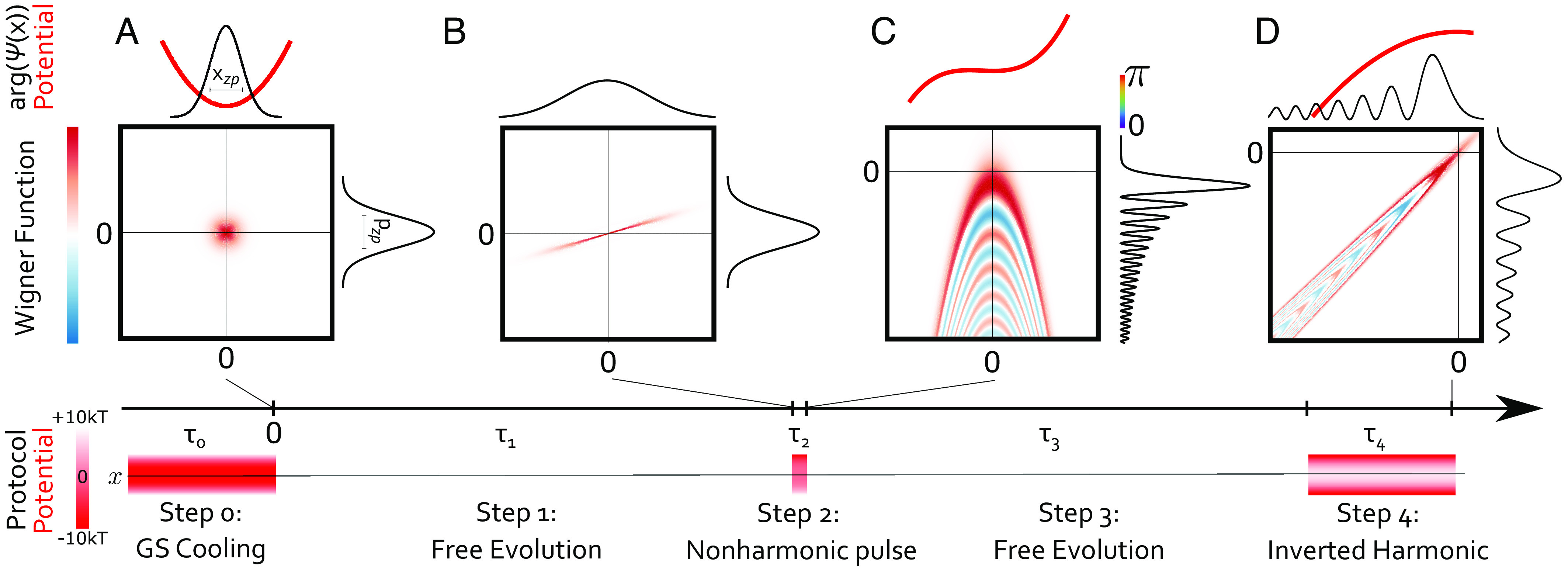
Preparation and imaging of a non-Gaussian state. The wavepacket of a single nanoparticle is initially prepared in the ground state of an harmonic potential (*A*). After a free expansion of the wavepacket of time $\tau_{1}$, (*B*) the particle briefly experiences a cubic and quadratic potential for time $\tau_{2}$ modulating the phase of the wavepacket while minimizing its momentum uncertainty resulting in a non-Gaussian Wigner function (*C*). We then image the corresponding momentum space fringe pattern into position space by a free evolution of time $\tau_{3}$. Finally, we enlarge the fringe spacing to a detectable size with an inverted potential (*D*).

The protocol consists of five steps (Fig. 1): 0) Initialization of the ground state in a harmonic potential, 1) free evolution, 2) pulsed interaction with a cubic potential, 3) free evolution, 4) evolution in an inverted potential. Finally, by switching back to the potential in step 0, the particle position is measured before initializing the state again. Note that in this protocol the particle practically stays at the same place and the process can be repeated immediately and with the same particle. In the following discussion, we do not presume any specific physical implementation until we discuss an optical setting later in the manuscript.

At the heart of the protocol is the preparation of a non-Gaussian state in step 2. The pulsed dynamics in a cubic potential $V_{2}\left(x\right)\propto x^{3}$ imprints a cubic phase on the particle wavefunction $\psi \left(x\right)\to \psi \left(x\right)\exp[-\left(i/\hbar \right)V_{2}\left(x\right)t]$ for a sufficiently short pulse duration that allows to neglect the kinetic term (for details see *Materials and Methods*; see also refs. 27 and 45 for alternative ideas to prepare non-Gaussian states using a cubic potential). In analogy to diffraction at a phase grating (12, 44, 46), the cubic phase results in fringes in momentum space, clearly reflecting its non-Gaussianity (Fig. 1*C*). For the fringes to form requires the preparation of a state with sufficient purity, large extension in position, and small momentum uncertainty. This is achieved via ground-state cooling in step 0 (Fig. 1*A*), a free evolution during step 1 (Fig. 1*B*), and introducing an additional quadratic potential during step 2. The fringe pattern can then be observed in position space after another free evolution (step 3) and subsequent expansion of the state (step 4, Fig. 1*D*).

We proceed with a more detailed description of each step. We consider the center-of-mass motion of a particle of mass *m* along one axis, say *x*. The protocol requires five piece-wise constant potentials $V_{i}\left(\hat{x}\right)$. The corresponding Hamiltonians are given by ${\hat{H}}_{i}={\hat{p}}^{2}/\left(2m\right)+V_{i}\left(\hat{x}\right)$, where $\hat{x}$ and $\hat{p}$ are the center-of-mass position and momentum operators with $[\hat{x},\hat{p}]=i\hbar$. In step 0, the potential is harmonic $V_{0}\left(x\right)=m\omega_{0}^{2}x^{2}/2$ with trap frequency $\omega_{0}$. In this step, during a time duration $\tau_{0}$, the particle is prepared in a thermal state with mean phonon occupation $\overset{¯}{n}<1$ and zero-point motion $x_{\text{zp}}\equiv {\left[\hbar /\left(2m\omega_{0}\right)\right]}^{1/2}$. In step 1, for a time duration $\tau_{1}\gg \omega_{0}^{-1}$, the potential is switched off, $V_{1}\left(x\right)=0$, to let the position uncertainty $\sigma_{x}\left(\tau_{1}\right)\approx x_{\mathrm{zp}}\sqrt{2\overset{¯}{n}+1}\omega_{0}\tau_{1}$ expand linearly in time, while the momentum uncertainty remains constant (Fig. 1*B*). In step 2, for a short time duration $\tau_{2}\ll 2m\hbar /\left\langle {\hat{p}}^{2}\right\rangle$ (appendix IV,2), the particle experiences the potential $V_{2}\left(x\right)=m\omega_{2}^{2}x^{2}/2+m\omega_{2}^{2}x^{3}/l$, where $\omega_{2}$ defines the stiffness of the harmonic potential and $\sigma_{x}/l$ quantifies the effect of the cubic potential. This is the key step in the protocol: The phase modulation of the cubic term creates sharp non-Gaussian features in momentum space (Fig. 1*C*) if the harmonic term sufficiently reduces the momentum uncertainty by canceling the quadratic phase acquired during step 1 ($\propto \tau_{1}^{-1}$). In step 3, for a time $\tau_{3}$, the potential is switched off, which maps momentum features into position space while linearly increasing their size. Note that the harmonic term in step 2 also allows to compensate for the quadratic phase ($\propto \tau_{3}^{-1}$) acquired during step 3. Altogether, after this step, the momentum features will be completely mapped into position space if $\omega_{2}^{2}\tau_{2}\approx \tau_{1}^{-1}+\tau_{3}^{-1}$ *SI Appendix*. In other words, we then expect a sharp image of the interference fringes. In step 4, for a time duration $\tau_{4}$, we apply an inverted harmonic potential $V_{4}\left(x\right)=-m\omega_{4}^{2}x^{2}/2$. It expands the position fringes exponentially fast (35, 47) (Fig. 1*D*) and allows to increase the fringe spacing beyond the achievable detection position resolution.

Assuming unitary evolution and a pure initial state, the position probability distribution at the end of the protocol (after step 4) is given by *SI Appendix*

[1] $$\begin{array}{c} P\left(x\right)=\frac{1}{\sqrt{2\pi }\Delta x^{2}\sigma_{c}}{\left[\mathrm{Ai}\left(\frac{x}{\Delta x}\right)*\exp\left(-\frac{x^{2}}{4\sigma_{c}^{2}}\right)\right]}^{2}, \end{array}$$

where ∗ denotes a convolution (with respect to *x*) of an Airy function $\mathrm{Ai}\left(x\right)$ (Δx determines the fringe spacing) with a Gaussian of width σ_*c*_. The detailed analytical derivation of Eq. **1** is given in *SI Appendix* including analytical expressions of the lengthscales Δx and σ_*c*_.

**Fig. 2.**
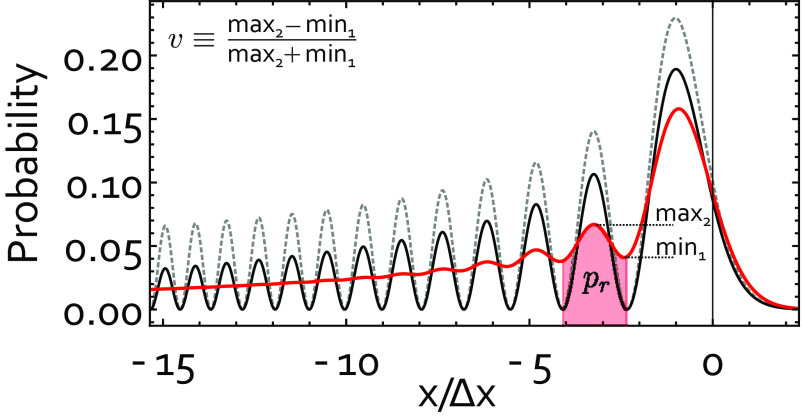
Interference pattern. Shown is the predicted probability distribution of detecting the nanoparticle at a position *x* at the end of a complete protocol run with (red) and without (black) decoherence, as well as the underlying square of the Airy function (dashed-gray). The plot is based on the parameters of our case study (Table 1). We use the visibility *v* and probability to observe the particle in the second fringe $p_{r}$ (shaded area) to define the valid fringe patterns in Fig. 3 (*SI Appendix*, section VII).

Fig. 2 illustrates the probability distribution Eq. **1** (black line) in comparison to the square of the pure Airy function (dashed gray line), which corresponds to the limit $\Delta x\gg \sigma_{c}$. Since here we coherently superimpose probability amplitudes, the visibility stays perfect even for finite σ_*c*_, yet the fringe height is exponentially reduced for increasing |x|. To observe multiple fringes, we require $\Delta x/\sigma_{c}>1$, which ensures that the fringe height is not reduced too much. One can determine this ratio in dependence on the protocol parameters as *SI Appendix*:

[2] $$\begin{array}{c} \frac{\Delta x}{\sigma_{c}}=2\sigma_{x}\left(\tau_{1}\right){\left[\frac{3m\omega_{2}^{2}\tau_{2}}{l\hbar }\right]}^{1/3}, \end{array}$$

**Table 1.** Parameter set for the case study

Initial conditions						Protocol parameters							Results			
$T_{e}\left[K\right]$	P[mbar]	λ[nm]	r[nm]	$\overset{¯}{n}$	$\omega_{0}\left[\text{kHz}\right]$	$\tau_{1}\left[\text{ms}\right]$	$\omega_{2}\left[\text{kHz}\right]$	$\phi_{2}$	$\tau_{2}\left[\mu s\right]$	$\tau_{3}\left[\text{ms}\right]$	$\omega_{4}\left[\text{kHz}\right]$	$\tau_{4}\left[\text{ms}\right]$	$T_{i}\left[K\right]$	$p_{r}$	*v*
300	$10^{-10}$	1,550	50	0.5	2π·100	1.34	2π·2.38	0.065π	10	0.66	2π·10	0.087	315.2	0.094	0.23

The interference pattern expected for this parameter set is shown in Fig. 2, red line. Properties of silica nano-particles: density $\rho =1,850\;{\text{kg/m}}^{3}$, heat capacity: $c_{v}=700$ J/K, refractive index at λ=1,550 nm: $n_{1,550}=1.43+\text{I}2.46\cdot 10^{-9}$.

with the position uncertainty $\sigma_{x}\left(\tau_{1}\right)$ after step 1 and a factor that incorporates the interaction strength with the cubic pulse in step 2. Since the latter is mainly limited by the added noise of the actual physical implementation, the condition $\Delta x/\sigma_{c}>1$ results in a condition on $\sigma_{x}\left(\tau_{1}\right)$.

A major limitation to the free expansion times is decoherence due to collisions with residual ambient gas molecules in the vacuum chamber, as a single collision resolves the particle position. To account for this, we limit the total protocol time $\tau_{f}\equiv \sum_{i=1}^{4}\tau_{i}$ such that no gas collision occurs in 90% of the protocol runs (*SI Appendix*, section V) and omit this source of decoherence in the further discussion. A second, universal source of decoherence is scattering, emission, and absorption of black-body radiation (48). It can be described with a master equation of the form

[3] $$\begin{array}{c} \dot{\rho }=-\frac{i}{\hbar }\left[{\hat{H}}_{i},\hat{\rho }\right]-\Lambda_{i}\left[\hat{x},\left[\hat{x},\hat{\rho }\right]\right]. \end{array}$$

Here, $\hat{\rho }$ is the density matrix of the center-of-mass state, and $\Lambda_{i}$ the localization rate. Both are assumed to be constant during each step *i* of the protocol. Depending on the actual implementation, the localization rates may also include other sources of decoherence (e.g., recoil heating).

The effect of decoherence as described by Eq. **3** and assuming an initially mixed Gaussian state can be calculated analytically. For $\Lambda_{1}x_{\mathrm{zp}}^{2}\tau_{1}\ll 1$, the final position probability distribution is given by *SI Appendix*, section II:

[4] $$\begin{array}{c} P_{D}\left(x\right)=\frac{P(x)}{\sqrt{2\pi \sigma_{\Lambda }^{2}}}*\exp\left(-\frac{x^{2}}{2\sigma_{\Lambda }^{2}}\right), \end{array}$$

which is a convolution of the position probability distribution obtained by the unitary dynamics in Eq. **1** with a Gaussian of variance $\sigma_{\Lambda }^{2}$ reducing the visibility of the interference fringes (as shown in Fig. 2, red line). The key parameter $\sigma_{\Lambda }$ depends on the localization rates during each of the protocol steps and the phonon occupation number $\overset{¯}{n}$ of the initial thermal state. Most importantly, for a finite visibility v>0, we require $\Delta x>\sigma_{\Lambda }$ (*SI Appendix*, Fig. S5).

For a further analysis, we need to be more specific about the physical implementation. Here, we consider a dielectric nanoparticle in a time-dependent potential that is realized with an optical standing wave: $V_{i}\left(x\right)=-\left(m\omega_{i}^{2}\lambda^{2}\right)/\left(8\pi^{2}\right)\cos^{2}\left(2\pi x/\lambda +\phi_{i}\right)$. The potentials $V_{i}\left(x\right)$ for each step can be obtained by controlling the laser intensity (proportional to $\omega_{i}^{2}$) and the phase $\phi_{i}$ of the standing wave (*SI Appendix*, section V) generating the harmonic (step 0: $\phi_{0}=0$), the harmonic + cubic (step 2: $0<\phi_{2}<\pi /8$) and the inverted (step 4: $\phi_{4}=\pi /2$) potential. We additionally assume electronic control of the charged particle for three purposes: feedback-based ground state cooling (20, 21), compensation of the linear part of the optical potential during step 2 (to keep the particle inside the trapping volume), and stabilization against gravity in the vertical direction.

As laser light exposure increases the internal temperature $T_{i}$ of the nanoparticle by absorption, $\Lambda_{1}$ and $\Lambda_{3}$ are dominated by black-body radiation (*SI Appendix*, section III), while $\Lambda_{2}$ and $\Lambda_{4}$ are dominated by scattering of laser photons (*SI Appendix*, section IV). Note that the internal heating during Step 2 and Step 4 is negligible compared to the heating during ground state cooling *SI Appendix*. For position detection, the optical field is switched back to step 0 and the first time period of detection $t_{D}\ll 2\pi /\omega_{0}$ can be used for position readout. Finite detection resolution will be compensated by the exponential increase of the size of the interference pattern in step 4. We design the protocol to confirm the existence of interference fringes with a 5σ confidence after approximately $1.2\cdot 10^{4}$ experimental runs, corresponding to less than 1 min total measurement time. This is achieved for $v^{2}p_{r}=0.005$ (*SI Appendix*, section VII/VIII; $p_{r}$ is defined in Fig. 2).

Creating quantum interference during step 2 requires the wave packet after step 1 to exhibit sufficient spatial coherence, where we quantify the coherence length $x_{c}$ as the width of the correlation function $g_{1}\left(x_{2}-x_{1}\right)=\;\exp\left(-{\left(x_{2}-x_{1}\right)}^{2}/\left(2x_{c}^{2}\right)\right)$, in full analogy to optical coherence (*SI Appendix*, section VI, 49). In turn, we can certify a lower bound on the coherence length before step 2 $x_{c}^{*}\left(\tau_{1}\right)\equiv 2\sigma_{x}\left(\tau_{1}\right)\sigma_{c}/\sigma_{\Lambda }<x_{c}\left(\tau_{1}\right)$ (*SI Appendix*, section VI) based on the experimental observation of the final shape of the interference pattern and on the width $\sigma_{x}\left(\tau_{1}\right)$ of the state after the first free expansion. For example, the observation of the probability density in Fig. 2 (red line) implies $x_{c}^{*}\left(\tau_{1}\right)\approx 713x_{\mathrm{zp}}\approx 6.6$ nm.

**Fig. 3.**
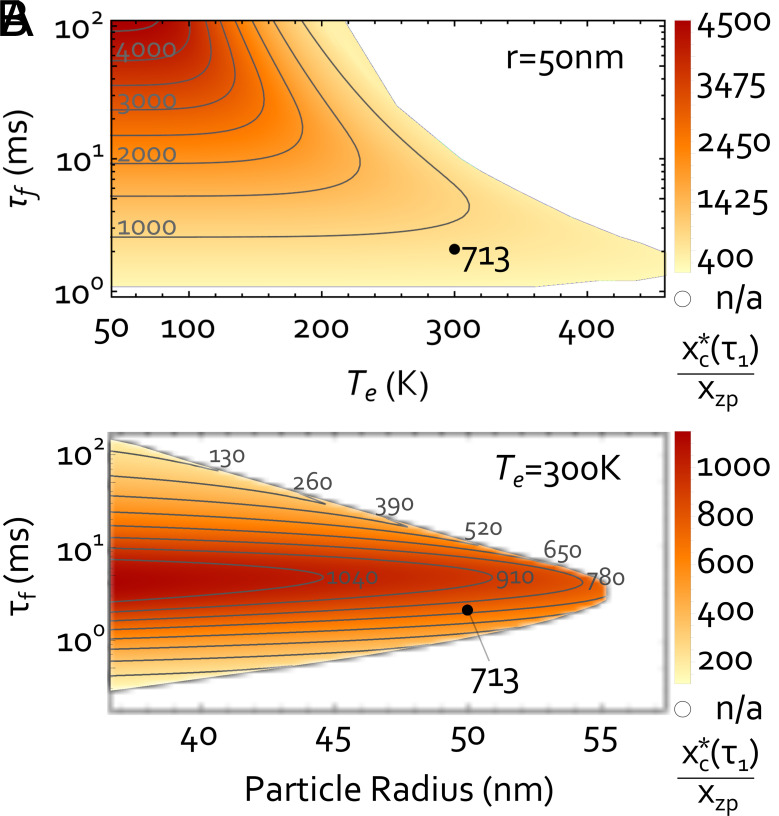
Certified coherence length: We show the lower bound on coherence length $x_{c}^{*}\left(\tau_{1}\right)$ that we can certify with our scheme in dependence on (*A*) the environmental temperature $T_{e}$ for a particle of r=50 nm and (*B*) the particle radius for $T_{e}=300$ K. The plots include the influence of decoherence from black-body radiation and photon recoil. The vertical axis shows the total protocol time $\tau_{f}$ for a single protocol run, respectively. We fix $v^{2}p_{r}=0.005$ and a fringe spacing of 5 nm for all data points (Details on optimization in *SI Appendix*, section VIII). In the white area, no combination of parameters produces an interference pattern with $v^{2}p_{r}\geq 0.005$.

In Fig. 3, we study the dependence of $x_{c}^{*}\left(\tau_{1}\right)$ on the particle radius *r*, environmental temperature $T_{e}$ and total protocol time $\tau_{f}$, effectively determining the pressure required for the experiment. The criteria on the interference correspond to those described for the case study and Fig. 2. See *SI Appendix*, section VIII, for details on the optimization. In Fig. 3*A*, we vary the particle radius *r* at room temperature ($T_{e}=300$ K); for Fig. 3*B*, we vary the environmental temperature $T_{e}$ for a radius of r=50 nm. In both panels, our case study is marked with a black dot. It can be seen that for our criteria, larger particle sizes are not accessible at room temperature and also superposition sizes are limited. Yet, cryogenic temperatures promise to enable the verification of much larger superpositions.

**Fig. 4.**
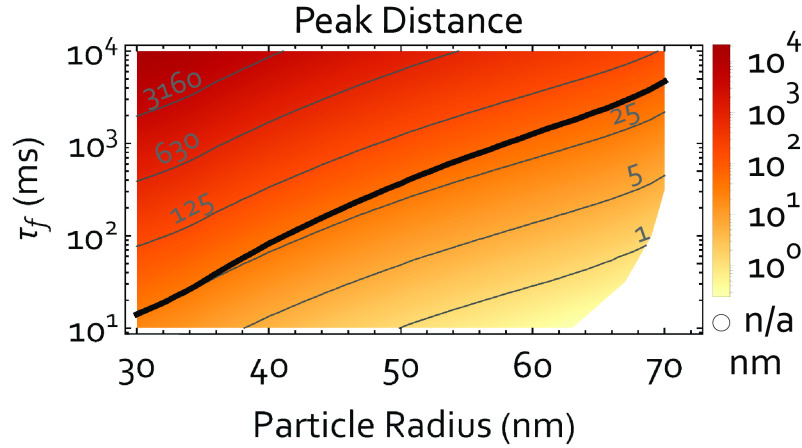
Coherent splitting: We show the distance between the first two probability maxima ($x_{\max1}$, $x_{\max2}$) of the interference patterns for protocols that preserve coherence between these maxima ($g_{1}\left(x_{\max2},x_{\max1}\right)>0.95$) for different particle radii *r* and protocol times $\tau_{f}$. Here, we do not include decoherence due to black-body radiation and omit step 4 (inverted potential). The black line shows the parameter set where the coherent splitting between the fringes corresponds to the particle diameter. In the white area, no combination of parameters produce an interference pattern with $g_{1}\left(x_{\max2},x_{\max1}\right)>0.95$.

Looking ahead, one may ask whether the optical cubic potential might also serve to coherently split the wavepacket, ideally even beyond the particle size. More precisely, under which conditions are the two main peaks in the interference pattern Fig. 2 coherent ($g_{1}\left(x_{\max1},x_{\max2}\right)>0.95$) and separated by more than the particle diameter ($|x_{\max1}-x_{\max1}|>2r$). To achieve this, we need to avoid the optical inverted potential in step 4 ($\tau_{4}=0$), as it localizes the state. Since without step 4, Δx grows only linearly in time (*SI Appendix*, section IX), this requires much longer protocol times $\tau_{f}$. To determine protocols for coherent splitting, we neglect decoherence/scattering rates due to black-body radiation/gas molecules in the calculation and note that they are bound by the inverse protocol time, respectively. We still assume $\overset{¯}{n}=0.5$. The result of this analysis is shown in Fig. 4. The analysis shows that optical control is compatible with a splitting of the particle wave packet even beyond the particle size (above the black line). The corresponding decoherence rates are very demanding and can likely only be achieved at cryogenic temperatures of the environment and the internal particle temperature. For example, for a particle of 50 nm radius, coherent splitting by 100 nm requires a coherence time of 1s. This can be achieved with a background pressure below $10^{-13}$ mbar and temperatures (of both environment and particle) of approximately 50 K (*SI Appendix*, section 8).

In summary, we presented a scheme to prepare and observe non-Gaussian features of a massive particle which is based on the control of pulsed potentials. We provided a platform-independent analytical analysis of our protocol. We theoretically showed that, despite of photon recoil, an optical implementation of our protocol can generate matter–wave interference for a silica nanoparticle with a mass exceeding ∼ 10^8^ a.m.u. at room temperature and with feasible vacuum levels. This significant reduction of environmental demands is enabled by operating at short lengths and timescales. We investigated how the generation of the interference pattern depends on the particle size and environmental temperature. In addition, we analyzed the extent of nanoparticle delocalization that is detectable via the interference pattern and characterized the environmental conditions that would be required to observe coherent splitting of the wavepacket beyond the particle size. We believe that the use of pulsed potentials provides a central element in the toolbox of levitated optomechanics, and, if successfully realized, with a significant impact on the field of macroscopic quantum physics.

## Materials and Methods

### Experimental Proposal: Optical Standing Wave.

As a specific implementation of our protocol, we discuss its feasibility using a dielectric nanoparticle in a time-dependent potential realized with an optical standing wave. In the following, we detail how this implementation allows for obtaining the required potentials. Then, we proceed by deriving the associated photon recoil localization rates along an optical standing wave. In three dimensional (3D), the normalized mode profile of an optical standing wave propagating in *x*-direction can be well approximated by $f\left(x,y,z\right)=\cos\left(kx-\phi \right)e^{-\left(y^{2}+z^{2}\right)/w^{2}\left(x\right)}$, where ϕ is the controlled phase of the standing wave and w(x) plays the role of an *x*-dependent beam waist. While the possibility of recoiled photons scattering into free space requires a full 3D analysis, it is sufficient to focus on the direction of propagation (*x*) in order to derive the required potentials to implement the protocol.

#### Optical potentials along a standing wave.

The electric field amplitude of a standing wave propagating in the *x*-direction can be written as

[5] $$\begin{array}{c} E\left(x\right)=E_{0}\cos\left(kx-\phi \right), \end{array}$$

which generates an optical potential for dielectric nano-particles of the form

[6] $$\begin{array}{c} V\left(x\right)=-\frac{m\omega^{2}}{2k^{2}}\cos^{2}\left(k\hat{x}-\phi \right), \end{array}$$

where ω is the mechanical frequency the particle experiences in the intensity maximum and serves us in the following as a measure for laser intensity. We obtain the required potentials for each step i=0,1,3,4 by controlling $\omega =\omega_{i}$ and the phase $\phi =\phi_{i}$, which stay constant during each step *i*. As the particle should stay inside the trapping volume inside which it can be re-captured and ground-state cooled, we propose to approximately compensate for arising linear optical potentials and the linear gravitational potential with an appropriate linear electric potential with the opposite sign. The center of mass wave function of the particle evolves with the general Hamiltonian

[7] $$\begin{array}{c} \hat{H}\left(\omega ,\phi \right)=\frac{{\hat{p}}^{2}}{2m}+F\left(\omega ,\phi \right)\hat{x}-\frac{m\omega^{2}}{2k^{2}}\cos^{2}\left(k\hat{x}-\phi \right), \end{array}$$

where $F\left(\omega ,\phi \right)\equiv \frac{m\omega^{2}}{2k}\sin\left(2\phi \right),$ is implemented by a linear electric potential. Here, we assume that noise associated with shot-to-shot imprecisions of optical and electric potentials can be neglected. In our case study, this is fulfilled if the relative precision of the respective optical pulse energy is $≲10^{-5}$ and if the SD on the positioning of the optical potentials relative to the trapping potential is ≲1pm *SI Appendix*. As ϕ and ω take different values for each step in our protocol, we shortly summarize the whole protocol:

- Step 0: $H_{0}=H\left(\omega_{0},0\right)$, $\tau_{0}=0$, optically trapped and ground-state cooled nano-sphere at an anti-node of a standing wave.
- Step 1: $H_{1}=H\left(0,0\right)$ free evolution for time $\tau_{1}$.
- Step 2: $H_{2}=H\left(\omega_{p},\phi_{2}\right)$ for time $\tau_{2}$ which describes a short optical standing-wave pulse, which to leading orders contains a quadratic and a cubic potential.
- Step 3: $H_{3}=H\left(0,0\right)$ second free evolution for time $\tau_{3}$.
- Step 4: $H_{4}=H\left(\omega_{4},\pi /2\right)$ describes a rapid expansion in an inverted harmonic potential for time $\tau_{4}$ by shifting the standing wave such that the particle is at a node of the standing wave.

As step 0 describes the well-known evolution in a harmonic potential, steps 1 and 3 are free evolution and step 4 generates the well-analyzed inverted harmonic potential (35, 47), we now focus on Step 2.

#### Short-lasting quadratic and cubic potential generated by optical standing wave pulse (step 2).

Expanding the potential in Eq. **7** until third order around x=0 for an arbitrary phase $\phi_{2}$ yields:

[8] $$\begin{array}{c} {\hat{H}}_{2}\left(\omega_{p},\phi_{2}\right)\approx \frac{1}{2}m\omega_{p}^{2}\cos\left(2\phi_{2}\right)x^{2}+\frac{1}{3}km\omega_{p}^{2}\sin\left(2\phi_{2}\right)x^{3}, \end{array}$$

where we dropped the constant part and assumed that the pulse is sufficiently short that we can neglect the kinetic term. Before we discuss the short-pulse condition, we want to focus on a couple of convenient properties of short pulses:

- As multiple subsequent short pulses commute with each other, the particle state does not depend on their order or if they are applied simultaneously. Thus, the quadratic and cubic pulse could also be applied separately in arbitrary order.
- Conveniently for experiments, only the pulse area matters (and not its shape): as H(x,t)≈V(x,t) commutes with itself at different times, the time evolution of a time-dependent short-pulse Hamiltonian can be written as
  [9] $$\begin{array}{c} \Psi (x,t)=\exp\left(-\frac{i}{\hbar }\int_{0}^{t}dt^{\prime }V(x,t^{\prime })\right)\Psi (x,0). \end{array}$$
  Since $\int_{0}^{t}dt^{\prime }V\left(x,t^{\prime }\right)$ is just proportional to the pulse area, we can in theory make the pulse arbitrarily short and compensate the short duration with a larger peak intensity without affecting the final state.

The last point enables us to always fulfill the conventional short-pulse requirement of $\left\langle {\hat{p}}^{2}\right\rangle \tau_{2}\ll 2m\hbar$, especially in the case of relatively small pulse areas (as we will see are optimal with respect to decoherence). However, in reality, the short-pulse approximation is much less restrictive if the short pulse is followed or proceeded by a free evolution, as the first-order correction is just an additional free evolution for $\tau_{2}$ (which can be added to the followed or proceeded free evolution time) and the next order correction is proportional to $\tau_{2}^{2}$. To connect the Hamiltonian at step 2 Eq. **8** to the general form given in the main text, we identify:

[10] $$\begin{array}{c} \omega_{2}^{2}=\cos\left(2\phi_{2}\right)\omega_{p}^{2} \end{array}$$

and

[11] $$\begin{array}{c} \frac{1}{l}=\frac{1}{3}k\tan\left(2\phi_{2}\right). \end{array}$$

## Supplementary Material

Appendix 01 (PDF)

## Data Availability

Mathematica scripts data have been deposited in Phaidra (https://phaidra.univie.ac.at/o:1646057) (50).
