# Supplementary material for: Fast quantum interference of a nanoparticle via optical potential control
Source: Proc Natl Acad Sci U S A. 2024 Jan 16;121(4):e2306953121. doi: 10.1073/pnas.2306953121 (PMC10823235; doi:10.1073/pnas.2306953121)
Supplement: Supplementary file 1 — Appendix 01 (PDF) [file pnas.2306953121.sapp.pdf]

Supporting Information for

Fast Quantum Interference of a Nanoparticle via Optical Potential Control

Lukas Neumeier, Mario A. Ciampini, Oriol Romero-Isart, Markus Aspelmeyer, Nikolai Kiesel

Mario A. Ciampini
E-mail: mario.arnolfo.ciampini@univie.ac.at

This PDF file includes:

Supporting text
SI References

Supporting Information Text

1. Protocol Without Decoherence

Here we derive Eq. (1) in the main text. As the initial state (step 0) we assume the ground state of a harmonic oscillator with mechanical frequency ω_0 and unitary evolution in steps 1,2,3 and 4. In this derivation, we omit normalization factors and normalize the end result.

Step 0: Groundstate Cooling. We begin with the groundstate of a harmonic oscillator

$$\Psi_0(x) \propto \exp\left(-\frac{x^2}{4x_{\text{zp}}^2}\right), \tag{1}$$

with zero-point motion $x_{\text{zp}} \equiv [\hbar/(2m\omega_0)]^{1/2}$.

Step 1: Free Evolution for τ_1. Using the free-evolution propagator we obtain:

$$\Psi_1(x) \propto \exp\left(-\frac{x^2}{4\sigma_x^2(\tau_1)} + \mathrm{i}\frac{\tau_1\omega_0}{4\sigma_x^2(\tau_1)}x^2\right), \tag{2}$$

with position variance $\sigma_x^2(\tau_1) = x_{\text{zp}}^2(1+\omega_0^2\tau_1^2)$.

Step 2: Cubic + Harmonic Pulse for τ_2. Since we assume this pulse to be sufficiently short (for requirements, see Methods), we neglect the kinetic part of the Hamiltonian $H_2 \approx V_2(x) = m\omega_2^2x^2/2 + m\omega_2^2x^3/l$ which results in multiplication with a quadratic + cubic phase:

$$\Psi_2(x) = \exp\left(-\frac{\mathrm{i}}{2\hbar}m\omega_2^2x^2\tau_2 - \frac{\mathrm{i}}{\hbar l}m\omega_2^2x^3\tau_2\right)\Psi_1(x). \tag{3}$$

Step 3 and 4: Free-Evolution for τ_3 followed by an inverted harmonic potential for τ_4. We again use the propagator for free-evolution (without solving the convolution-integral) and then the propagator for the evolution in an harmonic potential (1) while substituting the real mechanical frequency with an imaginary mechanical frequency $\mathrm{i}\omega_4$, which describes the evolution in an inverted harmonic potential. We solve the second integral first since it only involves Gaussians, obtaining

$$\Psi_4(x) \propto \exp\left(\mathrm{i}\frac{m\omega_4}{2\hbar}x^2\right)\int dx_1 \exp\left(-\frac{\mathrm{i}}{\hbar l}m\omega_2^2x_1^3\tau_2 - \frac{x_1^2}{4\sigma_x^2(\tau_1)} + \mathrm{i}b_cx_1^2 + \mathrm{i}\frac{2}{\hbar}\frac{m\omega_4\exp(-\omega_4\tau_4)}{1+\omega_4\tau_3}xx_1\right), \tag{4}$$

where we assumed $\omega_4\tau_4 \gg 1$ and with

$$b_c = \frac{m}{2\hbar}\left(\frac{\omega_4}{1+\tau_3\omega_4} + \frac{\tau_1\omega_0^2}{1+\omega_0^2\tau_1^2} - \omega_2^2\tau_2\right). \tag{5}$$

Using the convolution theorem in Eq. (4), yields

$$\Psi(x,\tau_4) \propto \exp\left(\mathrm{i}\frac{m\omega_4x^2}{2\hbar}\right)\left[\mathrm{Ai}\left(\frac{x}{\Delta x}\right) * \exp\left(-\frac{x^2}{4\sigma_c^2}\right) * \exp\left(\mathrm{i}\frac{x^2}{4\sigma_{\text{cb}}^2}\right)\right]. \tag{6}$$

Here $*$ denotes a convolution with respect to x and $\mathrm{Ai}\left(\frac{x}{\Delta x}\right)$ is the Airy-function, with

$$\frac{\Delta x}{\sigma_c} = 2\sigma_x(\tau_1)\left[\frac{3m\omega_2^2\tau_2}{\hbar l}\right]^{1/3}, \tag{7}$$

$$\sigma_c = \frac{\hbar}{4\sigma_x(\tau_1)}\frac{\omega_4\tau_3+1}{m\omega_4}e^{\omega_4\tau_4}, \tag{8}$$

and

$$\frac{\sigma_{\text{bc}}}{\Delta x} = \sqrt{\frac{b_c}{4}}\left[\frac{3m\omega_2^2\tau_2}{\hbar l}\right]^{-1/3}. \tag{9}$$

As the convolution with the quadratic phase reduces the visibility of interference fringes by lifting them off zero, we require $\sigma_{\text{bc}}/\Delta x < 1$. Then this convolution can be neglected as a convolution with oscillations on length scales much smaller than Δx average themselves out and the quadratic phase can be approximated by a delta function. This condition can be easily fullfilled by setting $b_c \approx 0$, which requires choosing

$$\omega_2^2\tau_2 = \frac{\omega_4}{1+\omega_4\tau_3} + \frac{\omega_0^2\tau_1}{1+\omega_0^2\tau_1^2} \approx \frac{1}{\tau_3} + \frac{1}{\tau_1}, \tag{10}$$

where we assume $\omega_0\tau_1 \gg 1$ and $\omega_4\tau_3 \gg 1$. Normalizing for $b_c \approx 0$ yields:

$$\Psi(x,\tau_4) = \frac{1}{(2\pi)^{1/4}\Delta x\sqrt{\sigma_c}}\exp\left(\mathrm{i}\frac{m\omega_4x^2}{2\hbar}\right)\left[\mathrm{Ai}\left(\frac{x}{\Delta x}\right) * \exp\left(-\frac{x^2}{4\sigma_c^2}\right)\right], \tag{11}$$

which absolute value square produces Eq. (1) in the main text. For completeness, we provide the first two moments in position and momentum:

$$\langle x(\tau_4)\rangle = -\frac{\Delta x^3}{4\sigma_c^2}, \quad [12]$$

and

$$\langle x^2(\tau_4)\rangle = \sigma_c^2\left(1+\frac{3\Delta x^6}{16\sigma_c^6}\right), \quad [13]$$

$$\langle p(\tau_4)\rangle = -\frac{m\omega_4\Delta x^3}{4\sigma_c^2}, \quad [14]$$

$$\langle p^2(\tau_4)\rangle = \frac{\hbar^2}{4\sigma_c^2}+\frac{3m^2\Delta x^6\omega_4^2}{16\sigma_c^4}+m^2\omega_4^2\sigma_c^2. \quad [15]$$

2. Position probability distribution for an initial thermal state subject to displacement noise

Here we derive Eq. (4) of the main text assuming as master equation of the form of Eq. (3) in the main text for each step and an initial Gaussian (e.g. thermal) state. Note that decoherence due to laser photons, black-body radiation, electric/magnetic field fluctuations and various linear white noise sources like linear shot/intensity noise can all be modeled by such a master equation (displacement noise) as long as the interacting particles cannot resolve the position of the particle better than its standard deviation. We separate the derivation into two parts:

- Before the non-linear interaction (before step 2), where the state is Gaussian and the effect of decoherence can be quantified by its size $\sigma_x(\tau_1)$ and the decay of its purity $P(\tau_1) = \mathrm{Tr}(\rho^2(\tau_1))$.
- During and after the non-linear interaction (during and after step 2), where $\sigma_x(\tau_1)$, $P(\tau_1)$ and decoherence/noise during step 2, 3 and 4 can be absorbed into a single total blurring distance σ_Λ, which reduces the visibility of the final interference peaks.

Initial thermal state (step 0) and displacement noise during the first free evolution (step 1). The time evolution of the Wigner function describing any Gaussian state evolving with a quadratic Hamiltonian can be expressed as (2)

$$W(x,p,t) = \frac{\sqrt{4a_1(t)a_2(t)-a_3^2(t)}}{2\pi}\exp\left(-a_1(t)x^2-a_2(t)p^2-a_3(t)px\right), \quad [16]$$

with

$$a_1(t) = \frac{2P^2(t)\sigma_p^2(t)}{\hbar^2}, \quad [17]$$

$$a_2(t) = \frac{2P^2(t)\sigma_x^2(t)}{\hbar^2} \quad [18]$$

and

$$a_3(t) = \pm\frac{2P^2(t)\sqrt{4\sigma_x^2(t)\sigma_p^2(t)-\hbar^2/P^2(t)}}{\hbar^2}, \quad [19]$$

where the sign of $a_3(t)$ is the sign of $\langle xp+px\rangle$, $\sigma_x^2(t)$ is the variance in position space and $\sigma_p^2(t)$ is the variance in momentum space. Thus, every Gaussian state is fully characterized by only three parameters (in the reference frame where $\langle x\rangle = \langle p\rangle = 0$).

With $P(0) = (2\bar{n}+1)^{-1}$, $\sigma_x^2(0) = x_{\mathrm{zp}}^2(2\bar{n}+1)$, $\sigma_p^2(0) = p_{\mathrm{zp}}^2(2\bar{n}+1)$, $x_{\mathrm{zp}} = \sqrt{\hbar/(2m\omega_0)}$, and $p_{\mathrm{zp}} = \sqrt{\hbar\omega_0 m/2}$, we specify our initial state $W(x,p,0)$ as a thermal harmonic oscillator with mechanical frequency ω_0 and average phonon occupation number $\bar{n}$. Note that for a thermal harmonic oscillator state $a_3(0) = 0$.

We proceed by calculating $P(\tau_1)$, $\sigma_x(\tau_1)$ and $\sigma_p(\tau_1)$ after the first free evolution (step 1) during which the particle experiences displacement noise (typically dominated by the emission of black-body radiation) with localization rate Λ_1. The time evolution of its Wignerfunction $W = W(x,p,t)$ is governed by (3)

$$\frac{\partial W}{\partial t} = -\frac{p}{m}\frac{\partial W}{\partial x}+\hbar^2\Lambda_1\frac{\partial^2 W}{\partial p^2}. \quad [20]$$

To solve this equation we switch the state representation to (2)

$$\tilde{W}(k_x,k_p,t) = \frac{1}{2\pi}\int dxdp\exp\left(-\mathrm{i}k_x x-\mathrm{i}k_p p\right)W(x,p,t), \quad [21]$$

which evolves with a simpler equation

$$\frac{\partial\tilde{W}}{\partial t} = -\frac{k_x}{m}\frac{\partial\tilde{W}}{\partial k_p}-\hbar^2\Lambda_1 k_p^2\tilde{W}. \quad [22]$$

We make a separation ansatz

$$\tilde{W} = \tilde{W}(k_x,k_p,t) = \tilde{W}_{\Lambda_1=0}(k_x,k_p,t)\tilde{G}(k_x,k_p,t), \quad [23]$$

where $\tilde{W}_{\Lambda_1=0}(k_x, k_p, t)$ describes free evolution without decoherence. $\tilde{G}(k_x, k_p, t)$ satisfies the same equation as $\tilde{W}$ with initial condition $G(k_x, k_p, 0) = 1$. Solving Eq. (22) for $G(k_x, k_p, 0)$ yields

$$\tilde{G}(k_x, k_p, t) = \exp\left(-\hbar^2\Lambda_1 t k_p^2 - \frac{\hbar^2\Lambda_1 t^2}{m} k_p k_x - \frac{\hbar^2\Lambda_1 t^3}{3m^2} k_x^2\right). \qquad [24]$$

To calculate the Wigner function after τ_1 we double inverse Fourier transform Eq. (23) back into the Wigner representation

$$W_1(x, p, \tau_1) = \frac{1}{2\pi}\int dk_x dk_p e^{\mathrm{i}k_x x + \mathrm{i}k_p p}\tilde{W}_{\Lambda_1=0}(k_x, k_p, \tau_1)\tilde{G}(k_x, k_p, \tau_1), \qquad [25]$$

where $\tilde{W}_{\Lambda_1=0}(k_x, k_p, \tau_1)$ is the double Fourier transform of $W(x - (p/m)\tau_1, p, 0)$.

After computing the Gaussian integrals of Eq. (25), we can identify $P^2(\tau_1)\sigma_x^2(\tau_1) = \hbar^2 a_2(\tau_1)/2$ with Eq. (18), where $a_2(\tau_1)$ is the coefficient of p^2 in the exponent. For $\omega_0^2\tau_1^2 \gg 1$ and $x_{\mathrm{zp}}^2\Lambda_1\tau_1 \ll 1$, we obtain

$$P(\tau_1) \approx \left(\frac{8}{3}\Lambda_1\tau_1\sigma_x^2(\tau_1) + (2\bar{n}+1)^2\right)^{-\frac{1}{2}}. \qquad [26]$$

We see how the purity gets reduced by displacement noise during a free evolution, while for $\omega_0^2\tau_1^2 \gg 1$ and $x_{\mathrm{zp}}^2\Lambda_1\tau_1 \ll 1$ the position uncertainty $\sigma_x(\tau_1)$ increases approximately linearly since

$$\sigma_x^2(\tau_1) = \int dp dx x^2 W(x, p, \tau_1) = x_{\mathrm{zp}}^2(2\bar{n}+1)(\omega_0^2\tau_1^2+1) + 2\Lambda_1\hbar^2\tau_1^3/(3m^2) \approx x_{\mathrm{zp}}^2(2\bar{n}+1)(\omega_0^2\tau_1^2+1), \qquad [27]$$

as does the momentum space variance

$$\sigma_p^2(\tau_1) = \int dp dx p^2 W(x, p, \tau_1) = p_{\mathrm{zp}}^2(2\bar{n}+1) + 2\Lambda_1\hbar^2\tau_1. \qquad [28]$$

These equations reproduce results previously obtained by (4).

Displacement noise during the cubic + harmonic pulse (step 2). As we now know the Gaussian state after step 1, we take it as our new initial state in the following derivation with corresponding values $a_1 \equiv a_1(\tau_1)$, $a_2 \equiv a_2(\tau_1)$ and $a_3 \equiv a_3(\tau_1)$. We begin by switching into the B-representation of quantum phase space (5) defined as

$$B(x, \Theta) = \int dp W(x, p) e^{-\mathrm{i}p\Theta}, \qquad [29]$$

leading to

$$B_1(x, \Theta) = \exp\left[-\left(a_1 - \frac{a_3^2}{4a_2}\right)x^2 - \frac{1}{4a_2}\Theta^2 - \mathrm{i}\frac{a_3}{2a_2}x\Theta\right], \qquad [30]$$

which evolves under the influence of displacement noise with localization rate Λ_2 with

$$\dot{B}(x, \Theta) = \left[\frac{-\mathrm{i}}{m}\frac{d^2}{dx d\Theta} + \frac{\tilde{V}}{\mathrm{i}\hbar} - \hbar^2\Lambda_2\Theta^2\right]B(x, \Theta). \qquad [31]$$

In step 2, $\tilde{V} = V^- - V^+ = -2\hbar u_2 x\Theta - 3u_3\hbar x^2\Theta - u_3\hbar^3\Theta^3/4$ since $V^\pm = V_2(x \pm \hbar\Theta/2)$, where $V_2(x) = u_2x^2 + u_3x^3$ describes the experienced potential at step 2. Aligned with our previous assumption of short pulses, we approximate $(d/dx)B(x, \Theta) = 0$. Thus,

$$B_2(x, \Theta, \tau_2) = \exp\left[-\frac{\mathrm{i}}{\hbar}\tilde{V}\tau_2 - \hbar^2\Lambda_2\Theta^2\tau_2\right]B_1(x, \Theta), \qquad [32]$$

and

$$W_2(x, p, \tau_2) = e^{-(a_1 - \frac{a_3^2}{4a_2})x^2}\int d\Theta \exp\left[\mathrm{i}\left(p + 3\mathrm{i}\tau_2u_3x^2 - \frac{a_3}{2a_2}x\right)\Theta - \left(\frac{1}{4a_2} + \frac{\sigma_2^2}{2}\right)\Theta^2 + \frac{\mathrm{i}}{4}\hbar^2\tau_2u_3\Theta^3 + 2\mathrm{i}\tau_2u_2x\Theta\right], \qquad [33]$$

with

$$\sigma_2^2 = 2\hbar^2\Lambda_2\tau_2, \qquad [34]$$

being the momentum blurring variance caused by displacement noise during step 2.

To see how an initial thermal state and decoherence during step 1 and 2 affects the final position probability distribution, we proceed by substituting the classical solutions of the Hamilton equations describing evolution during step 3 and 4 into the Wigner function. Thus, in order to account for the second free evolution (step 3), we replace x by $x - (\tau_3/m)p$ and then, in order to continue with the inverted potential in step 4, x by x_0 with

$$x_0 = x\cosh(\omega_4\tau_4) - \frac{p}{m\omega_4}\sinh(\omega_4\tau_4), \qquad [35]$$

and then all p by p_0 with

$$p_0 = p\cosh(\omega_4\tau_4) - m\omega_4 x\sinh(\omega_4\tau_4). \qquad [36]$$

Next, we take the coefficient in the exponent which is proportional to $p\Theta$, set it to zero, solve for u_2 which is given by

$$u_2 = -\frac{a_3}{4a_2\tau_2} + \frac{m\omega_4}{2\tau_2\tau_3\omega_4 + \tau_2\tanh(\omega_4\tau_4)}, \qquad [37]$$

and substitute the remaining u_2 with this expression in our current Wigner function Eq. (33). For $x_{\mathrm{zp}}^2\Lambda_1\tau_1 \ll 1$ and $\omega_0^2\tau_1^2 \gg 1$ we obtain $-\frac{a_3}{2a_2 m} \approx 1/\tau_1$, which with $u_2 = \omega_2^2 m/2$ and $\omega_4\tau_4 \gg 1$, leads to the condition

$$\omega_2^2\tau_2 \approx \frac{\omega_4}{\omega_4\tau_3 + 1} + \frac{1}{\tau_1}, \qquad [38]$$

which reproduces the condition for the decoherence free case. To obtain the position probability distribution, we integrate Eq. (33) over all p, which is a Gaussian integral. Note that all terms proportional to x^2 cancel and we are left with an integral proportional to

$$\langle x'|\rho_2(\tau_4)|x'\rangle \propto \int d\Theta \frac{1}{\sqrt{a_1 - \frac{a_3^2}{4a_2} - 3\mathrm{i}u_3\tau_2\Theta}} e^{\mathrm{i}\Theta x' + \mathrm{i}\frac{1}{4}\hbar^2\tau_2 u_3\Theta^3 - \left(\frac{1}{4a_2} + \frac{\sigma_2^2}{2}\right)\Theta^2}, \qquad [39]$$

where we substituted x with $x = \frac{\omega_4\tau_3+1}{m\omega_4}\cosh(\omega_4\tau_4)x'$ and where the index 2 of $\rho_2(\tau_4)$ indicates that we considered decoherence only up to and including step 2 so far. To proceed, consider the identity

$$\int d\Theta \frac{1}{\sqrt{\tilde{a}_1 - \mathrm{i}u_2\Theta}} e^{\mathrm{i}x\Theta - \frac{1}{4\tilde{a}_2}\Theta^2 + \mathrm{i}u_3\Theta^3} \propto \left|\mathrm{Ai}\left(\frac{x}{\Delta x}\right) * e^{-\frac{x^2}{2\hbar^2\tilde{a}_1}}\right|^2, \qquad [40]$$

if $\tilde{a}_2\tilde{a}_1 = 1/\hbar^2$, which resembles the Heisenberg limit for the harmonic oscillator ground-state. This identity can be proven by replacing a_1 with $\tilde{a}_1$, a_2 with $\tilde{a}_2$, setting $P(\tau_1) = 1$ and $\sigma_2 = 0$ in Eq. (39) which would then assume an initial ground-state while ignoring all sources of decoherence. Thus, we can equate it to Eq. (1) in the main text as both equations describe the exact same physics. Note that for an initial ground-state, $\tilde{a}_2 = (2x_{\mathrm{zp}}^2)^{-1}$. To turn Eq. (39) in a suitable form to use the above identity, we add and subtract a term of the form $\frac{1}{4}\hbar^2(a_1 - \frac{a_3^2}{4a_2})\Theta^2$ in the exponent. After using the convolution theorem, re-substituting x', and normalizing we obtain:

$$\langle x|\rho_2(\tau_4)|x\rangle = \frac{1}{2\pi\Delta x^2\sigma_{\Lambda 2}\sigma_c}\left|\mathrm{Ai}\left(\frac{x}{\Delta x}\right) * e^{-\frac{x^2}{4\sigma_c^2}}\right|^2 * e^{-\frac{x^2}{2\sigma_{\Lambda 2}^2}}, \qquad [41]$$

with Δx and σ_c given by equations (7) and (8) after replacing $\sigma_x^2(\tau_1)$ with $\sigma_x^2(\tau_1) = x_{\mathrm{zp}}^2(2\bar{n}+1)(\omega_0^2\tau_1^2+1)$. The blurring variance excluding decoherence effects after step 2 is now given by

$$\sigma_{\Lambda 2}^2 = \left[\left(\sigma_2^2 + \sigma_{01}^2\right)\left(\frac{\omega_4\tau_3+1}{m\omega_4}\right)^2\right]\frac{e^{2\omega_4\tau_4}}{4}, \qquad [42]$$

with the associated momentum space blurring variance due to an initial thermal state (step 0) subject to displacement noise during the first free-evolution (step 1)

$$\sigma_{01}^2 = \frac{\hbar^2}{4}\frac{1-P^2(\tau_1)}{P^2(\tau_1)\sigma_x^2(\tau_1)} = \frac{\hbar^2}{\sigma_x^2(\tau_1)}(\bar{n}+\bar{n}^2) + \frac{2}{3}\hbar^2\tau_1\Lambda_1. \qquad [43]$$

Next we calculate the contributions to the blurring distance due to displacement noise during step 3 and 4.

Displacement noise during Step 3 (second free-evolution) and during Step 4 (inverted potential). To derive the associated blurring distance due to displacement noise during step 3, we remember Eq. (25)

$$W_3(x,p,\tau_3) = \int dk_x dk_p e^{\mathrm{i}k_x x + \mathrm{i}k_p p}\tilde{W}_{3,\Lambda_3=0}(k_x,k_p,\tau_3)\tilde{G}(k_x,k_p,\tau_3), \qquad [44]$$

where $\tilde{W}_{3,\Lambda_3=0}(k_x,k_p,\tau_3)$ is the double Fourier transform of $W_2(x-(p/m)\tau_3,p,0)$, which is the Wigner function after the second free evolution including displacement noise during the first free evolution and displacement noise during step 2 but without displacement noise during the second free-fall as described by Eq. (33). To see how decoherence during the second free evolution translates into a blurring distance after step 4, we again replace x by x_0 with

$$x_0 = x\cosh(\omega_4\tau_4) - \frac{p}{m\omega_4}\sinh(\omega_4\tau_4), \qquad [45]$$

and then all p by p_0 with

$$p_0 = p\cosh(\omega_4\tau_4) - m\omega_4 x\sinh(\omega_4\tau_4). \qquad [46]$$

Then we integrate over all p since we are interested in the position probability distribution, and after integrating over k_p and changing variables $y = k_x/\cosh(\omega_4\tau_4)$ we obtain

$$\langle x|\rho_3(\tau_4)|x\rangle \propto \int dy e^{ixy}\tilde{W}_{\Lambda_3=0}(y\cosh(\omega_4\tau_4), \frac{y}{m\omega_4}\sinh(\omega_4\tau_4))\tilde{G}(y\cosh(\omega_4\tau_4), \frac{y}{m\omega_4}\sinh(\omega_4\tau_4)) \quad [47]$$

which after using the convolution theorem can be written as

$$\langle x|\rho_3(\tau_4)|x\rangle \propto \langle x|\rho_2(\tau_4)|x\rangle * e^{-\frac{x^2}{2\bar{\sigma}_3^2}}, \quad [48]$$

with

$$\bar{\sigma}_3^2 \approx \frac{2\hbar^2\Lambda_3\tau_3^3}{3m^2}\cosh^2(\omega_4\tau_4), \quad [49]$$

where we neglected the terms proportional to τ_3^2/ω_4 and τ_3/ω_4^2 as we assume $\omega_4\tau_3 \gg 1$. Thus, in order to account for displacement noise during the second free evolution we only have to add $\bar{\sigma}_3^2$ to Eq. (42) by exploiting the associative property of convolutions.

Displacement noise during step 4 is taken into account by another convolution with a Gaussian with associated blurring variance (6).

$$\bar{\sigma}_4^2 = \frac{\hbar^2\Lambda_4}{4m^2\omega_4^3}e^{2\omega_4\tau_4}, \quad [50]$$

which is valid for $\omega_4\tau_4 \gg 1$ and can be absorbed into the total blurring variance as well, which can be written as

$$\sigma_\Lambda^2 = \left[\left(\sigma_2^2 + \sigma_{01}^2\right)\left(\frac{\omega_4\tau_3 + 1}{m\omega_4}\right)^2 + \sigma_3^2 + \sigma_4^2\right]\frac{e^{2\omega_4\tau_4}}{4} + \sigma_5^2, \quad [51]$$

with

$$\sigma_3^2 = \frac{2\hbar^2\Lambda_3\tau_3^3}{3m^2}, \quad [52]$$

and

$$\sigma_4^2 = \frac{\hbar^2\Lambda_4}{m^2\omega_4^3}. \quad [53]$$

We also added σ_5^2 as the blurring variance due to the position detection itself, which depends on the resolution of the implemented detection scheme. Note that σ_5^2 can be neglected if $\Delta x \gg \sigma_5$. Since $\Delta x \propto \exp(\omega_4\tau_4)$, this limit can always be achieved for sufficiently large $\omega_4\tau_4$, which can be determined by solving $\Delta x \gg \sigma_5$ for $\omega_4\tau_4$ and largely depends on the implemented detection scheme. In our case study (parameters table I, main text) we obtain a fringe spacing of 5 nm for $\omega_4 = 2\pi \times 10$ kHz and $\tau_4 > 0.08$ ms, which is much larger than the detection resolution required during feedback based ground state cooling in our scheme. The final position probability distribution $P_D(x)$ (Eq. (4) in the main text) is obtained by replacing $\sigma_{\Lambda 2}$ by σ_Λ in Eq. (41).

While the first moments of this distribution are unaffected by noise, the second moments in position and momentum are increased by adding a term of σ_Λ^2 and $m^2\omega_4^2\sigma_\Lambda^2$, respectively. These terms can be obtained by first calculating the moments of $|\Psi(x,\tau_4)|^2$ in the momentum picture (i), then writing the second convolution in integral form, solving the resulting Gaussian integral first and exploiting the result from (i).

3. Black-body radiation

In most experimental realisations of the suggested protocol, the dominant decoherence mechanism during the free evolutions (step 1 and step 3) is the emission and absorption of black-body radiation, which is typically dominated by the internal temperature of the particle. The internal temperature increases due to the absorption of black-body radiation emitted by the walls of the vacuum chamber and absorbed laser photons during laser light exposure. However, the internal temperatures decreases during the free evolutions by emitting black-body radiation into the environment.

For the rest of this section, we assume an optical implementation. We begin by modeling the internal temperature of the particle during multiple experimental runs of our protocol. We assume that groundstate cooling is performed in the intensity maximum of an optical standing wave for a cooling time τ_0 leading to internal heating. Furthermore, we assume internal cooling during the rest of the protocol as most of the time the particle evolves freely without laser light exposure and only experiences negligible weak and short laser pulses with regard to the internal temperature of the particle during step 2 and 4.

The absorbed power from the trapping and cooling laser photons of a particle trapped in the anti-node of a standing wave can be derived to be (7, 8)

$$P_{\text{abs}} = 2\pi\epsilon_0 E_0^2 kcr^3 \text{Im}\frac{\epsilon_r(\omega_L) - 1}{\epsilon_r(\omega_L) + 2}, \quad [54]$$

where E_0 is the electric field amplitude of the standing wave, $k = 2\pi/\lambda$ is the wavevector, c the speed of light and $\epsilon_r(\omega_L)$ is the complex dielectric permittivity evaluated at the laser frequency ω_L. P_{abs} can be expressed in terms of the initial mechanical trapping frequency of the particle (7)

$$\omega_0 = \sqrt{\frac{3k^2\epsilon_0 E_0^2}{2\rho}\text{Re}\frac{\epsilon_r(\omega_L) - 1}{\epsilon_r(\omega_L) + 2}}, \quad [55]$$

where here ρ is the particle density, resulting in

$$P_{\text{abs}} = \frac{m\omega_0^2 c}{k}\beta, \tag{56}$$

with $\beta \equiv \text{Im}\frac{\epsilon_r(\omega_L)-1}{\epsilon_r(\omega_L)+2}/\text{Re}\frac{\epsilon_r(\omega_L)-1}{\epsilon_r(\omega_L)+2}$. We assume laser photons with a wavelength of $\lambda = 1550$ nm and use $\epsilon_r(\omega_L) = n_L^2$, with the complex refractive index of silica $n_L(1550\text{nm}) = 1.44 + \text{i}\,2.5 \times 10^{-9}$ (8).

With the internal particle energy $E_i = mc_m T_i$, where $c_m = 700\,\text{J/(kgK)}$ is the specific heat capacity of silica, the differential equation for the internal temperature of the particle is given by

$$mc_m \frac{dT_i}{dt} = P_{\text{abs}}(t) + Vp_{\text{bb}}(T_e) - Vp_{\text{bb}}(T_i), \tag{57}$$

where $p_{\text{bb}}(T)$ is the absorbed/emitted power per unit of volume V of a silica particle.

Its temperature dependency is numerically obtained by integrating the corresponding bulk measurement data (8) for $T > 100$ K followed by calculating the power emitted by a thermal dipole and extrapolating it for $T < 100$ K as shown in Fig. S1. To check our assumption that internal heating due to the exposure of the nanoparticle to the optical pulses in step 2 and 4 is negligible, we calculate the increase of the internal temperature of the particle due these pulses (ignoring the emission and absorption of black-body radiation in Eq. (57)):

$$\Delta T(\omega, \tau) = \frac{\omega^2 c}{c_m k}\beta\tau. \tag{58}$$

Here, ω is the associated trapping frequency of the intensity maximum of the standing wave and τ is the duration of exposure. We obtain $\Delta T(\omega_p, \tau_2) \approx 1.3 \cdot 10^{-6}$ K and $\Delta T(\omega_4, \tau_4) \approx 1.9 \cdot 10^{-4}$ K, well justifying our assumption to ignore these heating mechanisms.

Next, we assume that the particle has been trapped sufficiently long that it has reached a steady state internal Temperature T_{ss} before the first experimental run of the protocol, which is given by solving Eq. (57) for $T_i = T_{\text{ss}}$, $P_{\text{abs}}(t) = P_{\text{abs}}$ and setting $dT_{\text{ss}}/dt = 0$. For a trapping frequency of $\omega_0 = 2\pi \times 100$ kHz at $T_e = 300$ K, we obtain $T_{\text{ss}} \approx 329$ K. The reason why our calculated steady state temperature is much smaller as observed in optical tweezer traps is due to the much stronger curvature of the photon intensity mode profile (at the diffraction limit) in a standing wave setting. Thus, along a standing wave much lower laser powers are required to generate the same trapping frequencies as in optical tweezers.

The above steady state temperature becomes the initial condition to simulate N experimental runs with Eq. (57), where $P_{\text{abs}}(t)$ is a piecewise function which starts with 0 for time τ_f, then $P_{\text{abs}}(t) = P_{\text{abs}}$ for time τ_0, then $P_{\text{abs}}(t) = 0$ again for time τ_f and so on. Note that the internal temperature does not depend on the particle Volume V as all terms in Eq. (57) are proportional to V. The solution to Eq. (57) with initial condition $T_{\text{ss}} \approx 329$ K is shown in Fig. S2a) for $N = 50$ experimental runs. As can be seen, in average, the internal temperature cools off until it reaches a dynamical steady state of $T_i(\infty) \approx 315.2$ K after less than $N = 244$ experimental runs as shown in Fig. S2b), where heating during groundstate cooling and emission of black-body radiation during the free evolutions balance each other out. At this point the range of internal temperatures of the particle is just ≈ 0.2 K. Hence, we use the time independent $T_i(\infty)$ for the localization rates describing the emission of black-body radiation during the free evolution and suggest that the measurement data obtained from the first 244 experimental runs should not contribute to the measurement of the position probability distribution. Note that $T_i(\infty)$ can be calculated much faster with Eq. (57) by setting $dT_i/dt = 0$ and multiplying P_{abs} with a factor of $\tau_0/(\tau_0 + \tau_f)$ and solving for $T_i = T_i(\infty)$. This is a worst case approximation as the decohering effect of displacement noise scales with the position variance of the particle state, which is largest towards the end of the protocol, where the internal temperature drops below $T_i = T_i(\infty)$, as can be seen in Fig. S2b). Note that additional cooling time could in principle be added to the protocol if lower T_i are required.

Black-body localization rates. For the black-body localization rates during the free evolutions, the scattering of thermal photons can be neglected (4) and we are left with photon emission and absorption:

$$\Lambda_1 = \Lambda_3 = \Lambda_{\text{bb}} = V\gamma_{\text{bb}}(T_i(\infty)) + V\gamma_{\text{bb}}(T_e). \tag{59}$$

This expression can now be used to calculate the blurring distances σ_{01} (Eq. 43) during step 1 and σ_3 (Eq. 52) during step 3, which then get absorbed into the total blurring variance Eq. (51).

Note that, the extrapolation of $\gamma_{\text{bb}}(T)$ for $T < 100$ K is likely overestimating the actual localization rates since our extrapolation yields $\gamma_{\text{bb}}(T \to 0) \gg 1$, as can be seen in Fig. S1b).

4. Photon recoil decoherence along an optical standing wave (step 0, 2 and 4)

Here we derive the laser photon recoil induced localization rates in a optical-standing wave setup, as described in the Methods section of the main paper. $\Lambda_r(\phi, \omega)$ along the direction of propagation of a standing wave. The dominant interaction process for a sub-wavelength particle is Rayleigh scattering. Localization happens due to the scattering of standing wave photons, which carry information about the center-of-mass position away. The Lindblad term can be derived to be (9)

$$L(\rho) = \gamma_{\text{sca}} n_{\text{ph}} \left[\int d\Omega R(\vec{n}) f(\vec{r}) e^{-\text{i}k\vec{n}\cdot\vec{r}} \rho e^{\text{i}k\vec{n}\cdot\vec{r}} f^*(\vec{r}) - \frac{1}{2}\{|u(\vec{r})|^2, \rho\} \right], \tag{60}$$

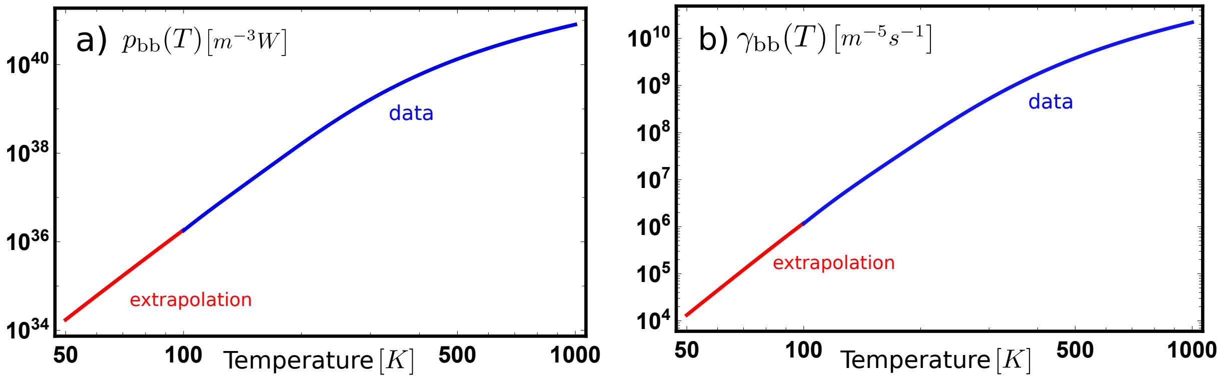

Fig. S1. Black-body radiation: emitted/absorbed power and localization rate of silica nano-spheres)
a) emitted/absorbed power $p_{\rm bb}(T)$ per unit volume as a function of temperature. Blue line is from integrating measurement data, red line is an extrapolation $p_{\rm bb}(T < 100\,K) \approx T^{-5.79}\exp\left(3.14\ln^2(T) - 0.265\ln^3(T)\right)$.
b) localization rate $\gamma_{\rm bb}(T)$ per unit volume as a function of temperature. Blue line is from relating the emitted power in a) to the momentum recoil that the particle receives from the leaving photons. The red line is an extrapolation $\gamma_{\rm bb}(T < 100\,K) \approx 1.91 \times 10^{31} T^{8.38}\ \exp\left(-0.19\ln^2(T)\right)$.

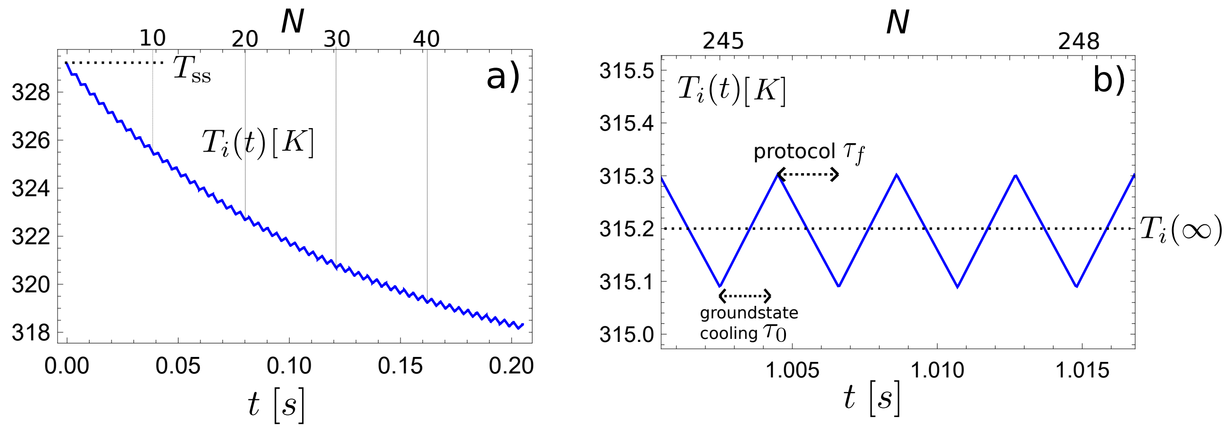

Fig. S2. Internal Temperature $T_i(t)$: Case Study

a) Internal Temperature $T_i(t)$ as a function of time (lower x-axis) and number of experimental runs N (upper x-axis) for a standing wave trapping frequency of $\omega_0 = 2\pi \times 100$ kHz at room temperature $T_e = 300$ K, cooling time of $\tau_0 = 2$ ms and protocol time $\tau_f = 2.1$ ms

b) The Internal Temperature $T_i(t)$ with same parameters as in a) reaches a dynamical steady state $T_i(\infty) \approx 315.2$ K after less than $N = 245$ experimental runs.

with the single photon Rayleigh scattering rate $\gamma_{\text{sca}} = ck^4 \text{Re}\left[\alpha(\omega_L)\right]^2/(6\pi\epsilon_0^2 V_m)$, the particle polarizability $\alpha(\omega_L)$, the number of photons $n_{\text{ph}} = E_0^2\epsilon_0 V_m/(2\hbar\omega_L)$, the mode volume V_m, the laser frequency ω_L, the normalized mode profile $f(\vec{r}) = \cos(kx-\phi)e^{-(y^2+z^2)/w^2(x)}$, and the emission pattern of a radiating dipole $R(\vec{n}) = \frac{3}{8\pi}(1-\sin^2(\Theta)\cos^2(\Phi))$ (here we assume the incident laser light is linearly polarized along the y-axis). Assuming a motional state with a width $\sigma_{\text{x,y,z}} \ll \lambda$, we approximate $w(x) \approx w(x_0) = \text{const}$ and expand the terms $f(\vec{r})e^{\pm \text{i}k\vec{n}\cdot\vec{r}}$ until linear order around $x=0$, $y=0$, and $z=0$, which allows us to perform the integral with $d\Omega = \sin(\Theta)d\Phi d\Theta$:

$$\int d\Omega R(\vec{n}) f(\vec{r}) e^{-\text{i}k\vec{n}\cdot\vec{r}} \rho e^{\text{i}k\vec{n}\cdot\vec{r}} f^*(\vec{r}) \approx \beta_x(\phi) x\rho x + \beta_y(\phi) y\rho y + \beta_z(\phi) z\rho z + O\rho, \quad [61]$$

where the term $O\rho$ is cancelled by a part of the last term of Eq. (60), which maintains the Lindblad form of the master equation. Note that all cross-terms of the form $x\rho y$ vanish after integration. The pre-factors are given by

$$\beta_x(\phi) = \frac{1}{10}k^2(7-3\cos(2\phi)) \quad [62]$$

$$\beta_y(\phi) = \frac{1}{5}k^2\cos^2(\phi) \quad [63]$$

$$\beta_z(\phi) = \frac{2}{5}k^2\cos^2(\phi). \quad [64]$$

Note that $\beta_x + \beta_y + \beta_z = k^2$, implying that the total energy increase due to photon recoil does not dependent on ϕ. However, the distribution of contributions to the localization rates for the different coordinates depends on the particle position along the standing wave. If the particle is at an anti-node ($\phi = 0$), we reproduce the well known distribution of localization rates of 1/5 (along polarization axis), 2/5 (orthogonal to direction of propagation and polarization axis), 2/5 (direction of propagation) for a far-detuned atom trapped in the intensity maximum of a standing wave (10). Counter-intuitively, for a particle in a node $\beta_x(\pi/2) = k^2$ is maximal, while $\beta_y(\pi/2) = \beta_z(\pi/2) = 0$, consistent with the fact that in a node the quadratic potential orthogonal to the direction of propagation vanishes. The photon recoil localization rates along a standing wave can be expressed as

$$\Lambda_r(\phi,\omega) = \frac{\pi^2\omega^2\rho V^2}{5\hbar\lambda^3}\text{Re}\left[\frac{\epsilon_r(\omega_L)-1}{\epsilon_r(\omega_L)+2}\right](7-3\cos(2\phi)), \quad [65]$$

where ω is the trapping frequency the particle would experience at an anti-node ($\phi=0$) and which reproduces rates previously calculated for a particle actually trapped at an anti-node (7). Thus, for step 2, $\Lambda_2 = \Lambda_r(\phi_2,\omega_p)$, where ϕ_2 and ω_p remain to be optimized and for step 4, $\Lambda_4 = \Lambda_r(\pi/2,\omega_4) = \frac{2\pi^2\omega_4^2\rho V^2}{\hbar\lambda^3}\text{Re}\left[\frac{\epsilon_r(\omega_L)-1}{\epsilon_r(\omega_L)+2}\right]$. These expressions can now be used to specify the previously calculated blurring distances σ_2 (Eq. (34)) and σ_4 (Eq. (53)), which are then absorbed into the total blurring distance σ_Λ (Eq. (51)).

5. Gas Collisions

Since during our protocol the thermal de-Broglie wavelength $\lambda_{\text{th}} = \sqrt{2\pi\hbar^2/(m_g k_b T_e)}$ of the background gas molecules with mass m_g and temperature T_e is much smaller than the position width of the particle state σ_x, a single gas collision localizes the state. Thus, to have a negligible effect of gas collisions on the measured interference pattern, most experimental runs should be completely free of gas collisions. Here, we first derive the probability $\mathcal{P}_0(T_e, r, P, \tau_f)$ of having no gas collisions during an experimental run lasting τ_f, which depends on the particle size r, the temperature T_e and the pressure P inside the vacuum chamber. In the second part we provide the required pressures $P_{0.9}$ such that 90% of experimental runs are free of gas collisions which could resolve the position x or transfer momentum along the x-direction. Assuming a Maxwell-Boltzmann distribution for the velocities of the gas particles, we arrive at an average collision rate of

$$\gamma_g = \frac{8\pi P r^2}{m_g\langle v_g\rangle}, \quad [66]$$

where P is the pressure in the vacuum chamber and $\langle v_g\rangle = \sqrt{8k_b T_e/(\pi m_g)}$ is the mean velocity of the Maxwell-Boltzmann distribution of the gas molecules. Assuming gas-particle collisions are statistically independent, rare and γ_g does not depend on time, it can be shown that the collisions follow Poissonian statistics and the probability of n gas collisions during the time τ_f is given by (11)

$$\mathcal{P}_n(T_e, r, P, \tau_f) = \frac{(\gamma_g\tau_f)^n}{n!}e^{-\gamma_g\tau_f}. \quad [67]$$

As we are only interested in the x-direction, the probability of no gas collisions with a momentum-component along this single spacial degree of freedom is

$$\mathcal{P}_x(T_e, r, P, \tau_f) = e^{-\gamma_g\tau_f/3}, \quad [68]$$

where we assume that the scattering rate along the x-direction is $\gamma_g/3$. Since we operate in ultra high vacuum, we assume a gas composition dominated by H_2 molecules with $m_g = 2$ a.m.u. Furthermore, we fix $\mathcal{P}_x(T_e, r, P_{0.9}, \tau_f) = 0.9$, ensuring that 90% of experimental runs are gas collision free along x. Fig. S3a) shows the required pressures $P_{0.9}$ in order to ensure

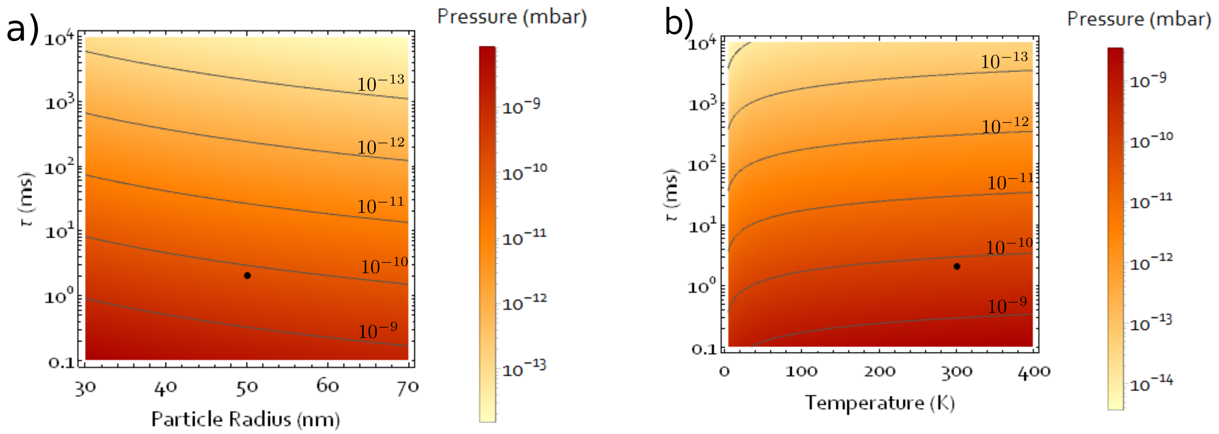

Fig. S3. Required Pressure $P_{0.9}$ to ensure that 90% of experimental runs are gas collision free along the x-direction by enforcing $\mathcal{P}_x(Te, r, P_{0.9}, \tau_f) = 0.9$.
a) Required pressure $P_{0.9}$ as function of particle radius r and total protocol time τ_f for a fixed environmental temperature $T_e = 300$ K.
b) Required pressure $P_{0.9}$ as function of environmental temperature T_e and total protocol time τ_f for a fixed particle radius $r = 50$ nm.
In both plots we assume that the gas composition is dominated by H_2 molecules with $m_g = 2$ u. The black dot represents our case study, which requires a pressure of $P_{0.9} \approx 1.4 \times 10^{-10}$ mbar.

$\mathcal{P}_x(T_e = 300K, r, P_{0.9}, \tau_f) = 0.9$ as a function of particle radius r and protocol time τ_f in a room temperature environment. Fig. S3b) shows the required pressures $P_{0.9}$ in order to ensure $\mathcal{P}_x(T_e, r = 50\text{nm}, P_{0.9}, \tau_f) = 0.9$ as a function of environmental temperature T_e by fixing the particle radius to $r = 50$ nm. The black dot in both plots represents our case study where we assume a silica particle with $r = 50$ nm, which requires a pressure of $P_{0.9} \approx 1.4 \times 10^{-10}$ mbar at room temperature in order experience no gas collisions in 90% of experimental runs with $\tau_f \approx 2.1$ ms.

6. Coherence: Quantification and Certification

As metric for the degree of coherence between two arbitrary positions x_1 and x_2 of a given particle density matrix $\rho_{\text{ij}} = \langle x_i|\rho|x_j\rangle$ we use the first order correlation function $g_1(x_1, x_2, t)$ (12) by replacing electric fields with density matrix elements at a given moment in time:

$$g_1(x_1, x_2, t) = \frac{\rho_{12}(t)}{\sqrt{\rho_{11}(t)\rho_{22}(t)}}, \quad [69]$$

where in the case of $\rho_{11} = \rho_{22}$ (equal amplitudes), $|g_1(x_1, x_2)|$ is the visibility of an interference pattern created with a perfect double-slit experiment (infinitely narrow slits, located at x_1 and x_2, no added decoherence). For a Gaussian state,

$$|g_1(x_1, x_2, t)| = \exp\left(-\frac{(x_1 - x_2)^2}{2x_c^2(t)}\right), \quad [70]$$

is just another Gaussian. With a given purity $P(t) = \text{Tr}(\rho^2(\text{t}))$ at time t and position width $\sigma_x(t)$, we call the length-scale

$$x_c(t) = \frac{2P(t)\sigma_x(t)}{\sqrt{1 - P^2(t)}}, \quad [71]$$

over which the first order correlation function decays (its standard deviation) "coherence length" (in analogy with optical coherence), which quantifies the spatial distance over which positions are predominantly in a coherent superposition (have a fixed phase relationship) and could in principle destructively interfere with each other. This definition implies that for positions $|x_2 - x_1| > x_c$ (which are further apart than x_c), the first order correlation function $g_1(x_1, x_2) < \exp(-0.5) \approx 0.6$.

Interestingly, the contribution σ_{01}^2 to the total blurring variance σ_Λ^2 caused by an impure state $P(\tau_1) < 1$ after the first free evolution (step 1) is just inversely proportional to $x_c^2(\tau_1)$ times the square of the classical momentum to position mapping function as can be seen in Eq. (43), where $\sigma_{01}^2 = \hbar^2/x_c^2(\tau_1)$. One can extract σ_Λ by de-convolving the final interference pattern until the result has maximum visibility and a lower bound on $x_c(\tau_1)$ can be found by pessimistically assuming that all blurring was caused by a finite $x_c(\tau_1)$ at step 1. Thus, $x_c(\tau_1) > \frac{\hbar}{\sigma_\Lambda} x_{\text{map}}(t)$, where $x_{\text{map}}(t)$ is the classical momentum to position space mapping function. However, with a cubic phase-grating protocol it is possible to certify a lower bound on $x_c(\tau_1)$ without knowing $x_{\text{map}}(t)$ by also extracting σ_c from the final interference pattern and measuring the position variance $\sigma_x^2(\tau_1)$ after step 1. Calculating σ_c/σ_Λ, the classical mapping function cancels and we obtain:

$$x_c(\tau_1) > 2\sigma_x(\tau_1)\frac{\sigma_c}{\sigma_\Lambda}. \quad [72]$$

Note that for this result we don't need to know any additional experimental parameters.

7. 5σ confirmation of the particle wave-nature: A Toy-Model

Here, we address the confidence we can have in observing the wave nature of the particle based on our measurement data. The signature of detection is the observation of an interference pattern, which implies the existence of a local minimum surrounded by two interference peaks. We focus on the difference in detection events between the minimum surrounded by the first and the second largest interference maximum (see Fig 2 in the main text). For this toy model, we only address the difference in detection events between the *second* largest peak and the minimum. We will now estimate the experimental runs (ensemble size) required to confirm their difference in detection events with $m \times \sigma$ confidence. A more sophisticated evaluation will likely reduce the number of required experimental runs.

To simplify the estimation, we assume that the relevant region (second largest peak and first minimum) of the final interference pattern $P_D(x)$ (Fig. 2 and Eq. (4) in main text) can be approximated by a full period of a sinusoidal function

$$f(x) = \frac{1}{2}(\max - \min)\sin(x) + \frac{1}{2}(\max + \min), \quad [73]$$

which is illustrated in Fig. S4.

Here, we absorb probability normalization into the values of max and min and only care about the end result and its scalings. The probability $p_r = p_{\max} + p_{\min}$ of finding the particle at the relevant positions (area below $f(x)$) is divided into two detection slots $p_{\max}$ and $p_{\min}$, which are the probabilities of detecting a particle contributing to the maximum or minimum, respectively, per experimental run. For N experimental runs, the number of detection events in each slot are then given by $n_{\max} \approx N \int_0^\pi dx f(x)$ and $n_{\min} \approx N \int_\pi^{2\pi} dx f(x)$. To confirm the existence of a minimum with $m \times \sigma$ confidence, the difference

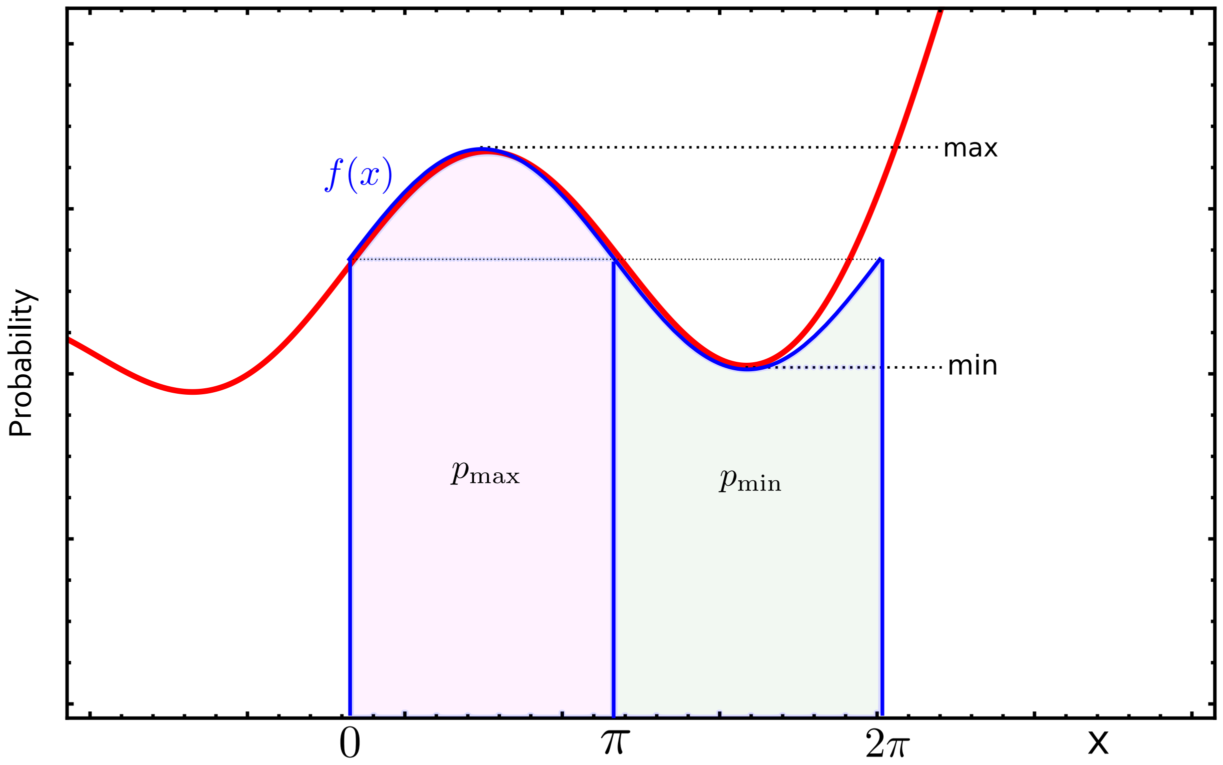

Fig. S4. Experimentally distinguishing a minimum from a maximum. The relevant area of the final interference pattern $P_D(x)$ (red) shown in Fig. 2 (main text) is assumed to be approximately described by a sinusoidal function $f(x)$ (blue, Eq. (73)), such that a single oscillation captures the relevant region $0 \leq x \leq 2\pi$. We divide the relevant area $p_r \approx \pi(\max + \min) = p_{\max} + p_{\min} = \int_0^{2\pi} dx f(x)$ into two slots, where $p_{\max}$ and $p_{\min}$ are the probabilities of finding the particle in the area which contributes to the maximum and minimum, respectively.

in detection events inside each slot should be m times larger than the combined standard deviation of each slot, which is mathematically expressed with the condition

$$n_{\max} - n_{\min} \gtrapprox m\sqrt{\sigma_{\max}^2 + \sigma_{\min}^2}, \qquad [74]$$

with statistical uncertainties $\sigma_{\max}^2 = n_{\max}$ and $\sigma_{\min}^2 = n_{\min}$. With the definition of the visibility $v = (\max - \min)/(\max + \min)$, we arrive at the condition

$$N \gtrapprox \frac{\pi^2}{4}\frac{m^2}{v^2 p_r}, \qquad [75]$$

in order to obtain a $m \times \sigma$ confirmation of the particle wave-nature. To conveniently apply this formula to Eq. (4) in the main text, we use (13)

$$\left|\mathrm{Ai}\left(\frac{x}{\Delta x}\right) * \exp\left(-\frac{x^2}{4\sigma_c^2}\right)\right|^2 \propto \left|\mathrm{Ai}\left(\frac{x}{\Delta x} + \frac{\sigma_c^4}{\Delta x^4}\right)\right|^2, \qquad [76]$$

note that the subsequent convolution with $\exp\left(-x^2/(2\sigma_\Lambda^2)\right)$ does not affect the location of the minima and maxima and then numerically calculate the corresponding maxima and minima of $\mathrm{Ai}^2(x)$. Then,

$$\max \approx P_D(-3.248\Delta x - \sigma_c^4/\Delta x^3), \qquad [77]$$

and

$$\min \approx P_D(-2.338\Delta x - \sigma_c^4/\Delta x^3). \qquad [78]$$

Furthermore, p_r is roughly the area below the second largest peak:

$$p_r \approx \int_{x_{\min 2}}^{x_{\min 1}} dx P_D(x), \qquad [79]$$

with

$$x_{\min 2} \approx -4.088\Delta x - \sigma_c^4/\Delta x^3, \qquad [80]$$

and

$$x_{\min 1} \approx -2.338\Delta x - \sigma_c^4/\Delta x^3, \qquad [81]$$

being the two minima surrounding the second-largest peak. For Fig. 3 and Fig. 4 in the main text, we fix $N \approx 1.2 \cdot 10^4$ and $m = 5$ for each data point allowing different combinations of v and p_r which can be optimized for any specific goal.

8. Optimization of experimental parameters for Fig. 3 and Fig. 4

Here we describe a fast way to find the optimal combination of experimental parameters ω_i, t_i and ϕ_2 which maximize the lower bound on certified coherence length $x_c(\tau_1)$ (Fig. 3) or coherent peak distance $\propto \Delta x(\tau_3)$ (Fig. 4). In both figures, we set a "quality-standard" for the final interference pattern, which we enforce for all data points. We achieve this by roughly fixing the number of experimental runs required to have a 5σ confirmation of matter-wave interference, which comes down to fixing the quality-parameter

$$q \equiv v^2 p_r \approx (5\pi/2)^2 N_{5\sigma}^{-1} \qquad [82]$$

for each data point in the figures.

A. Enforcing a quality standard. Remarkably, writing x in Eq. (4) (main text) in units of Δx leads to an equation, which only depends on two parameters: $p_c \equiv \sigma_c/\Delta x$ and $p_\Lambda \equiv \sigma_\Lambda/\Delta x$. We call these two parameters the "shape-parameters" since their values completely determine the shape and thus also the visibility v, relevant area p_r and therefore the quality $q = v^2 p_r$ of the final interference pattern. However, different values of p_c and p_Λ can lead to the same quality q since a lower visibility v can be compensated by a larger relevant area p_r (or vice versa). Their optimal combination depends on the metric we wish to maximize.

We enforce the chosen quality-standard q by restricting the search space to shape-parameters p_c and p_Λ which satisfy the contour-equation

$$q(p_c, p_\Lambda) = v^2 p_r \approx (5\pi/2)^2 N_{5\sigma}^{-1} = \text{const}, \qquad [83]$$

where we fix $N_{5\sigma}$ as we desire. In Fig. S5 (left), we plot the shape-quality q as a function of p_c and p_Λ, which span up the shape-space, which is the space of all possible shapes. On the right, we show three examples of possible shapes for $q = 0.005$ (black line) corresponding to $N_{5\sigma} \approx 1.2 \cdot 10^4$. Once we decide on a wished shape quality, we numerically solve the contour-equation (83) for

$$p_\Lambda^{(q)}\left[p_c\right], \qquad [84]$$

which tells us the required p_Λ for a given p_c to meet our quality-standard q.

B. Enforcing the condition Eq. (10). Enforcing the condition (10) allows us to eliminate $\omega_2^2\tau_2$ in p_c and p_Λ by replacing it with $\tau_1^{-1} + \tau_3^{-1}$, where we assume $\omega_4\tau_3 \gg 1$. Note that in p_Λ, the product $\omega_2^2\tau_2$ appears in σ_2 (Eq. (34)) after replacing Λ_2 with Eq. (65) and ω_p^2 with $\omega_2^2 = \cos 2\phi_2 \omega_p^2$.

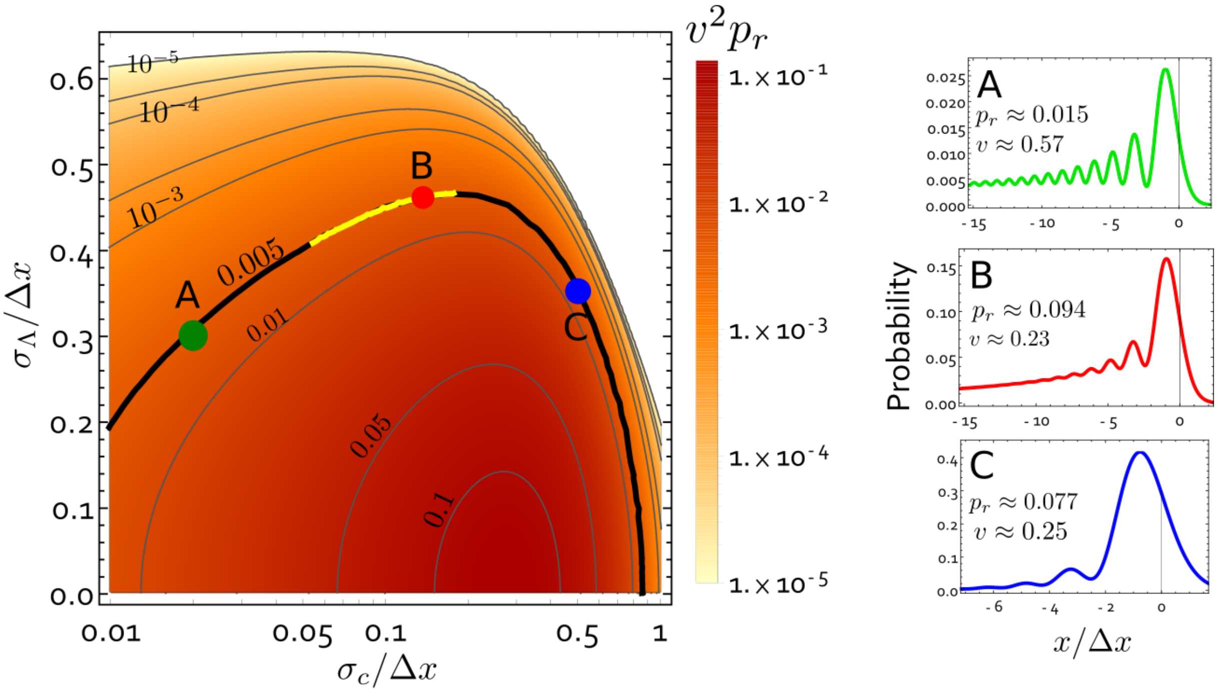

Fig. S5. Shape-Space: The space of possible shapes of interference patterns.
Left: Shape-quality $q = v^2 p_r$ as a function of shape-parameters $p_c \equiv \sigma_c/\Delta x$ and $p_\Lambda \equiv \sigma_\Lambda/\Delta x$. In the white region $v = 0$. The black contour fixes $q = 0.005$, which corresponds to $N_{5\sigma} \approx 1.2 \cdot 10^4$. Points A, B and C are example shapes shown on the right side with their corresponding values of p_r and v. The region around B, representing our case study with $p_c \approx 0.14$, tolerates the largest amount of noise (quantified by σ_Λ). The yellow line on top of the black line shows all possible accessible shapes for our case study with a fixed total protocol time of $\tau_f \approx 2.1$ ms. To produce the other possible shapes, ϕ_2 and τ_1 need to be chosen accordingly, while the other parameters are the same as in the example parameter set (table 1) in the main text. Point B maximises the lower bound of $x_c(\tau_1)$ of all accessible shapes. All optimal combinations of parameters for Fig. 3 and 4 in the main text are roughly found between A and B as for both plots large $\sigma_c/\Delta x$ lead to lower values of $x_c(\tau_1)$ or Δx, respectively.

C. Choosing values for ω_0 and ω_4. As quality $q = v^2 p_r$ monotonically increases with larger values of ω_0 and ω_4, these values are constrained by experimental boundary conditions. Here we choose $\omega_0 = 2\pi \times 100$ kHz to an experimentally realistic initial trapping frequency compatible with previous ground-state cooling experiments (14–17). However, larger values of ω_0 significantly decrease fringe blurring caused by a finite thermal occupation (Eq. (43)), allowing us to tolerate larger initial $\bar{n}$.

For $\omega_4 \tau_3 \gg 1$, ω_4 only appears in the blurring variance $\sigma_4^2 \propto \omega_4^{-1}$. Here, we fix $\omega_4 = 2\pi \times 10$ kHz as the blurring caused by the cubic pulse is roughly $\omega_4 \tau_3$ larger than the blurring caused by the inverted potential and further increasing ω_4 does not significantly increase q. This choice implies $\tau_3 \gg 0.016$ ms to satisfy the assumption $\omega_4 \tau_3 \gg 1$.

D. Enforcing a fixed fringe distance in Fig. (3) (with inverted potential). In Fig. (3) (main text), all data points have the same distance between the first two interference minima $\approx 1.75 \Delta x = 5$ nm. Remarkably, the inverted potential effectively decouples the shape (p_c,p_Λ) from the size $\propto \Delta x$ of the final interference pattern, since p_c and p_Λ do not depend on τ_4, while $\Delta x \propto \exp(\omega_4 \tau_4)$. Hence, as τ_4 has no effect on shape and quality, we fix the distance between the first two interference minima by simply solving the equation $1.75\Delta x = 5$ nm for τ_4 after finding the optimal combination of the remaining free parameters, which are τ_1, τ_3 and ϕ_2.

E. Maximizing the lower bound on coherence length $x_c(\tau_1)$ for Fig. 3 in the main text. Now, p_c is a function of τ_1, τ_3, ϕ_2 and r, while p_Λ is a function of τ_1, τ_3, ϕ_2, r, T_e and T_i. The total protocol time is dominated by the two free evolutions $\tau_f \approx \tau_1 + \tau_3$ (which is on the order of milliseconds) since $\tau_4 \sim 0.1$ ms and τ_2 can be chosen arbitrarily short as only pulse area counts (here we choose $\tau_2 = 10^{-2}$ ms). Thus, for Fig. 3, we first calculate $T_i = T_i(\infty)$ for a chosen τ_f and T_e with Eq. (57) by choosing a groundstate cooling time of $\tau_0 = 2$ ms. Then, we fix a particle radius r and numerically find the combinations of τ_1 (note that $\tau_3 \approx \tau_f - \tau_1)$) and ϕ_2 which solve the contour-equation

$$p_\Lambda^{(q)} \left[p_c(\tau_1, \phi_2) \right] = p_\Lambda(\tau_1, \phi_2)], \qquad [85]$$

for a given r, τ_f and T_e, which guaranties our chosen quality standard. The range of possible values of p_c and p_Λ with these found combinations of τ_f and ϕ_2 for $\tau_f \approx 2.1$ ms, are drawn into Fig. S5 (yellow line around point B), where the remaining parameters are as in the example parameter set in the main text. Next, we plug all found combinations of τ_1 and ϕ_2 (if there are any) into the equation for the lower bound of $x_c(\tau_1)$ (Eq. (72)) and pick the combination which yields the largest value (Point B in Fig. S5). All optimal shapes for Fig. 3 in the main text are roughly found between A and B as large $\sigma_c/\Delta x$ and simultaneously small $\sigma_\Lambda/\Delta x$ imply a small τ_1. A small τ_1 requires large $\omega_2^2 \tau_2$ to meet the condition (10), which increases $\sigma_\Lambda/\Delta x$ leading to lower values of $x_c(\tau_1)$.

With the combination of τ_1 and ϕ_2, which maximizes the lower bound of $x_c(\tau_1)$, we calculate the required τ_4 in order to have 5 nm fringe spacing as explained in section D. On the y-axis in Fig. 3 we show $\tau_f = \tau_1 + \tau_2 + \tau_3 + \tau_4$ and plot the largest found value of $x_c(\tau_1)$. In Fig. 3a) we fix $r = 50$ nm and scan T_e for the x-axis, while in Fig. 3b) we fix $T_e = 300$ K and scan r for the x-axis.

F. Maximizing coherent peak distance for Fig. 4 in the main text. First we calculate $g_1(x_{\max1}, x_{\max2}, \tau_3)$ (defined in the next section) for all combinations of p_c and p_Λ which satisfy the contour-equation (83) for $N_{5\sigma} \approx 1.2 \cdot 10^4$. Since for all combinations $g_1(x_{\max1}, x_{\max2}, \tau_3) > 0.95$, we conclude that our applied quality-standard implies coherence of > 0.95 between the two largest peaks as long as additional decoherence during the second free evolution can be neglected. Then we do the exact same thing as in the previous section but instead of picking the largest $x_c(\tau_1)$, we pick the largest peak distance $\approx 2.23\Delta x(\tau_3)$ from the found combinations of τ_1 and ϕ_2 which satisfy Eq. (85). Here we replace Δx with $\Delta x(\tau_3)$, σ_c with $\sigma_c(\tau_3)$ and σ_Λ with σ_{Λ_3}, which are defined in the following section.

To estimate the requirements on temperature (black body radiation decoherence) and pressure (gas collision decoherence) based on the timescale of the protocol (y-axis of Fig. 4), we can interpret the inverse timescale as an upper bound on the allowed decoherence rates. For our case study and splitting on the order of the size of the nanoparticle, this results in the following numbers: For the pressure we find $p \approx 10^{-13}$ mbar using equation (68). For black-body radiation decoherence using equations (26),(59), and (71), allows a worst case approximation for the coherence length of a Gaussian state after a free evolution of 1s. For $T_e = T_i = 50K$ we obtain $x_c(t = 1s) \approx 300$nm. Accordingly for a splitting of $x_c \approx 2r = 100$ nm we conclude that coherence persists between the two largest peaks.

9. Coherent Splitting without inverted potential

Here we are interested in the coherence between individual peaks of the final position probability distribution. We skip step 4 (inverted potential), as we estimate the requirements on localization rates in order to maintain significant coherence is hard or impossible to achieve with existing technology. However, clean electric potentials might change that in the future. We consider an optical standing wave implementation in a cryogenic environment and thus only consider photon recoil decoherence and an initial thermal state. The requirements on pressure and temperature can then be extracted from the total protocol time by calculating the corresponding gas scattering rates and black-body localization rates. To quantify the coherence between different positions, we now proceed by calculating the first-order correlation function

$$g_1(x_1, x_2, \tau_3) = \frac{|\langle x_1 | \rho(\tau_3) | x_2 \rangle|}{\sqrt{\langle x_1 | \rho(\tau_3) | x_1 \rangle \langle x_2 | \rho(\tau_3) | x_2 \rangle}}, \qquad [86]$$

after τ_3 (after step 3). It is convenient to substitute $x_1 = x + x'/2$ and $x_2 = x - x'/2$, as arbitrary density matrix elements of $\rho(\tau_3)$ can be calculated with

$$\langle x + \frac{x'}{2}|\rho(\tau_3)|x - \frac{x'}{2}\rangle = \frac{1}{h}\int dp e^{\mathrm{i}x'p/\hbar} W_2(x - (p/m)\tau_3, p), \tag{87}$$

where $W_2(x - (p/m)\tau_3, p)$ is the Wigner function (Eq. (33)) after step 2, where we replaced all x with $x - (p/m)\tau_3$. Note that in order for complete position space mapping, we again require $\omega_2^2\tau_2 \approx \tau_1^{-1} + \tau_3^{-1}$. The Gaussian integral over p can be solved analytically and the remaining θ integration can be solved numerically for $x' \neq 0$. For $x' = 0$, the final position probability distribution can be written as

$$\langle x|\rho(\tau_3)|x\rangle = \frac{1}{2\pi\Delta x^2(\tau_3)\sigma_{\Lambda 2}(\tau_3)\sigma_c(\tau_3)}\left|\mathrm{Ai}\left(\frac{x}{\Delta x(\tau_3)}\right) * \exp\left(-\frac{x^2}{4\sigma_c^2(\tau_3)}\right)\right|^2 * \exp\left(-\frac{x^2}{2\sigma_\Lambda^2(\tau_3)}\right), \tag{88}$$

with

$$\Delta x(\tau_3) = \hbar\left(\frac{km}{\hbar}\tan(2\phi_2)\omega_2^2\tau_2\right)^{1/3}\frac{\tau_3}{m}, \tag{89}$$

$$\sigma_c(\tau_3) = \frac{\hbar}{2\sigma_x(\tau_1)}\frac{\tau_3}{m}, \tag{90}$$

and

$$\sigma_\Lambda(\tau_3) = \sqrt{\sigma_2^2 + \frac{\hbar^2}{\sigma_x^2(\tau_1)}(\bar{n} + \bar{n}^2)}\frac{\tau_3}{m}. \tag{91}$$

Fig. S6 shows $g_1(x, x_{\max 2}, \tau_3)$ (black), with $x_{\max 2}$ being the position of the second largest peak, as a function of an arbitrary position x revealing the oscillating decay of coherence with distance from the second largest peak following the shape of the interference pattern at τ_3 (red). Remarkably, the interference peaks maintain strong coherence with each other, while coherence between minima and maxima almost vanishes. For Fig. 4 in the main text, we maximize $\Delta x(\tau_3)$ while requiring $g_1(x_{\max 1}, x_{\max 2}, \tau_3) > 0.95$ and $N \approx 1.2 \cdot 10^4$ experimental runs to confirm the wave-nature of the particle with 5σ confidence.

10. Experimental stability requirements

Here we study experimental requirements on the suppression of additional noise sources like external vibrations, laser phase noise, electric stray fields and the required precision on optical and electric pulse areas. In the end of this section, we provide upper limits on these noise sources for our case study (parameters of Table I in the main text).

A. External vibrations and laser phase noise. Ground-state cooling a nano-particle with feedback inside an optical trap requires suppression of phase and vibrational noise to a level below noise coming from the scattering of photons (photon recoil). Here, we assume a similar level of control for the optical pulses during the protocol's step 2 and 4, and neglect phase and vibrational displacement noise compared to photon recoil during these optical pulses. However, phase noise and external vibrations can cause small drifts in the position of the optical standing wave potential during ground-state cooling (step 0) and during the free evolution (step 1 and 3). This causes the particle to experience slightly different optical potentials along x in each experimental run. Additionally, experimental imprecision in generating the correct standing wave's phase shift will contribute to random shifts of the experienced potentials. In Fig. S7a) we illustrate how we model the random potential shifts by introducing the stochastic variables δx_i, $(i = 2, 4, 5)$ for each optical potential at step i with expectation values $\langle\delta x_i\rangle = 0$ and standard deviations $\sigma_{\delta \mathrm{x_i}}$. We also assume that all δx_i follow a Gaussian distribution and are statistically independent. Phase shift errors between the trapping potential (step 0) and the readout pulse (step 5) only accumulate during the duration τ_f of a single experimental run as the origin of our coordinate system is "reset" to the trapping potential after each experimental run. Since the interference pattern after step 4 is of the size of tens of nanometers, with fringe distances of 5 nm, we can neglect the small random shifts δx_5 of the read-out potential. As those shifts (as we will see below) must be of the order of picometers, they are well below the required resolution σ_5 of the read-out pulse.

Thus, we can define the relative shifts δx_i of the optical potentials with respect to the trapping potential (Fig. S7a)), which serves as the origin of the coordinate system for each experimental run. We proceed by calculating the associated blurring distances (which reduce the visibility of the final interference pattern) as a function of the standard deviation $\sigma_{\delta \mathrm{x}}$ of these random shifts for each relevant step in the protocol. With these results, we plot the visibility reduction of our case study as a function of this standard deviation in Fig. S7a).

A.1. Step 2: cubic + harmonic pulse. During the first free evolution external vibrations and phase noise cause a random drift of δx_2 in the position of the potential at step 2. The dominant stochastic part (which is the linear part, as higher orders are suppressed by factors of $k\sigma_x(\tau_1)$) of this potential $\tilde{V}_2$ is approximately proportional to δx_2 (expand Eq. (7) in the main text with $x \to x + \delta x_2$ to linear order in δx_2 and x):

$$\tilde{V}_2(x) \approx m\omega_p^2\cos(2\phi_2)\delta x_2 x. \tag{92}$$

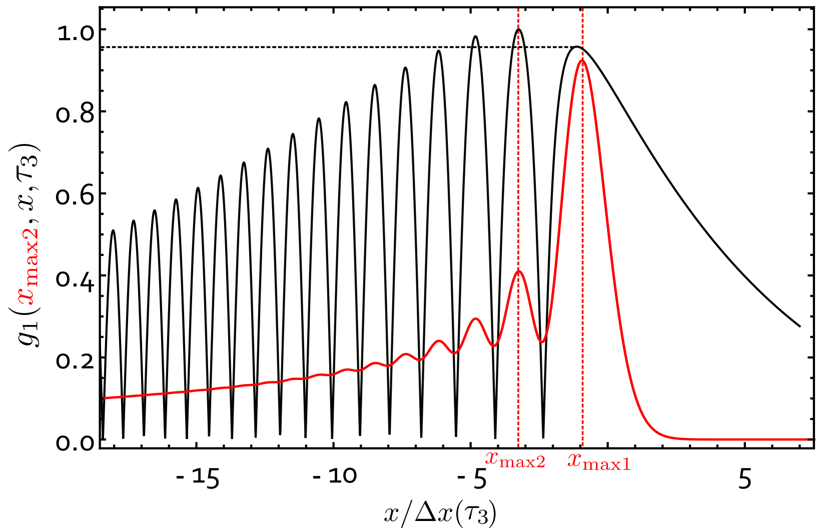

Fig. S6. Coherence between interference peaks. Shown is $g_1(x_{\max2}, x, \tau_3)$ (black) as a function of x, which quantifies coherence between the second largest peak $x_{\max2}$ and an arbitrary position x. For $x = x_{\max1}$ (the largest peak), we observe $g_1(x_{\max1}, x_{\max2}, \tau_3) \approx 0.96$, which is indicated by the horizontal dotted black line. The interference pattern (red) corresponds to Fig. 4 in the main text, with peak distance $x_{\max2} - x_{\max1} \approx 2.23\Delta x(\tau_3) = r = 50$ nm which requires a total protocol time of $\tau_f \approx 350$ ms with $\tau_1 \approx 0.92$ ms, $\tau_3 \approx 349$ ms, $\phi_2 \approx 0.9\pi/4$, $\omega_2 \approx 2\pi \times 1.66$ kHz. We assume $\bar{n} = 0.5$. The probability of finding the particle within the two largest peaks is ≈ 0.31.

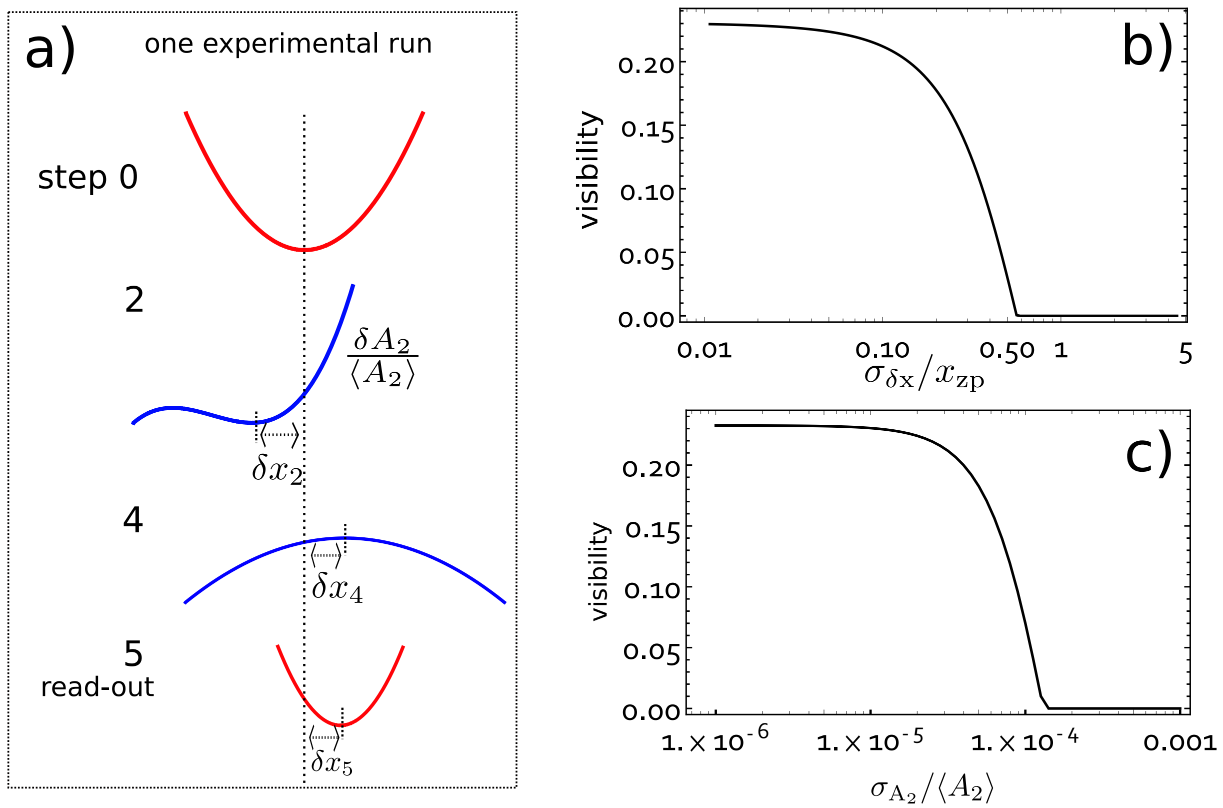

Fig. S7. Experimental stability

a) Shown is an example of a single experimental run with randomly shifted potentials relative to the trapping potential at step 0. These random shifts are modeled by the stochastic independent variables δx_i at step $i = 2, 4, 5$. Additionally, the precision error on the pulse area (with expectation value $\langle A_2 \rangle$) of the optical pulse at step 2 is modeled by the stochastic variable δA_2 with $\langle \delta A_2 \rangle = 0$ and standard deviation σ_{A_2}.

b) Case study: Visibility as a function of the standard deviation of δx_i. Here we take the ensemble average of the Gaussian distributed variables δx_i (while ignoring δA_2), assume that they all have the same standard deviation $\sigma_{\delta x}$, and plot the visibility of the final interference pattern of our case study (table I main text) as a function of that standard deviation.

c) Case study: Visibility as a function of relative precision error $\sigma_{A_2}/\langle A_2 \rangle$ on the pulse area with expectation value $\langle A_2 \rangle$ of the optical pulse during step 2 of the protocol. In this plot, we ignore δx_i and only take the ensemble average over the Gaussian distributed stochastic variable δA_2. As we propose an electric field pulse to cancel the linear part of the optical potential, we obtain the same behaviour for the relative precision error on the pulse area of the electric field pulse during step 2.

Since the pulse is assumed to be sufficiently short to neglect the kinetic evolution, we multiply the integrand of Eq. (4) with the term $\exp(-\mathrm{i}\tilde{V}_2(x_1)\tau_2/\hbar)$ and set $b_c = 0$ (note that this condition is not affected by linear noise):

$$\Psi_4(x) \propto \exp\left(\mathrm{i}\frac{m\omega_4}{2\hbar}x^2\right)\int dx_1 \exp\left(-\frac{\mathrm{i}}{\hbar l}m\omega_2^2 x_1^3\tau_2 - \frac{x_1^2}{4\sigma_x^2(\tau_1)} + \mathrm{i}\tilde{x}x_1\right), \quad [93]$$

with

$$\tilde{x} = \frac{2m\omega_4\exp(-\omega_4\tau_4)}{\hbar(1+\omega_4\tau_3)}x - \frac{m}{\hbar}\omega_p^2\tau_2\cos(2\phi_2)\delta x_2. \quad [94]$$

After using the convolution theorem we arrive at

$$\Psi_4(x) \propto \exp\left(\mathrm{i}\frac{m\omega_4}{2\hbar}x^2\right)\mathrm{Ai}\left(\frac{x-y}{\Delta x}\right) * \exp\left(\frac{(x-y)^2}{4\sigma_c^2}\right), \quad [95]$$

with $y \equiv \omega_p^2\tau_2(1+\omega_4\tau_3)\cos(2\phi_2)\delta x_2(2\omega_4)^{-1}\exp(\omega_4\tau_4)$. Next we take the absolute value squared of the last equation and perform the Gaussian weighted ensemble-average over the stochastic variable δx_2 in order to obtain the final position probability distribution:

$$P(x) \propto \int dy \exp\left(-\frac{y^2}{2\sigma_{\mathrm{v}2}^2}\right)\left[\mathrm{Ai}\left(\frac{x-y}{\Delta x}\right) * \exp\left(\frac{(x-y)^2}{4\sigma_c^2}\right)\right]^2 \propto \left[\mathrm{Ai}\left(\frac{x}{\Delta x}\right) * \exp\left(\frac{x^2}{4\sigma_c^2}\right)\right]^2 * \exp\left(-\frac{x^2}{2\sigma_{\mathrm{v}2}^2}\right), \quad [96]$$

where we exploited the definition of a convolution. Thus, a randomly shifted potential during step 2, where the random shifts have a standard deviation of $\sigma_{\delta \mathrm{x}_2}$, results in an additional convolution with a Gaussian with blurring distance

$$\sigma_{\mathrm{v}2} = \frac{\omega_p^2\tau_2(1+\omega_4\tau_3)\cos(2\phi_2)}{2\omega_4}\exp(\omega_4\tau_4)\sigma_{\delta \mathrm{x}_2}. \quad [97]$$

A.2. Step 4: Inverted Potential. The inverted harmonic potential at step 4 subject to fluctuations of its positioning x is given by

$$V_4(x) = -\frac{1}{2}m\omega_4^2(x-\delta x_4)^2, \quad [98]$$

with stochastic and Gaussian distributed variable δx_4 with $\langle\delta x_4\rangle = 0$ and standard deviation $\sigma_{\delta \mathrm{x}_4}$. The stochastic linear potential during step 4 reads:

$$\tilde{V}_4(x) \approx -m\omega_4^2 x\delta x_4. \quad [99]$$

Evolution in an inverted plus such a potential is described by the propagator for a quadratic plus a linear potential

$$K(x,x',t) \propto \exp\left[-\frac{m\omega}{2\mathrm{i}\hbar\sin(\omega t)}\left(\left(x^2+x'^2\right)\cos(\omega t) - 2xx'\right) - \frac{ma(\cos(\omega t)-1)}{\omega\mathrm{i}\hbar\sin(\omega t)}\left(x+x'\right)\right], \quad [100]$$

by replacing $\omega = \mathrm{i}\omega_4$ and using the stochastic acceleration $a = -\omega_4^2\delta x_4$. Using this propagator for step 4 in the derivation of $\Psi_4(x)$ in section 1, and continuing with a similar calculation as in section A.1, yields another convolution with a Gaussian of the final interference pattern with associated blurring distance

$$\sigma_{\mathrm{v}4} = \frac{1}{2}e^{\omega_4\tau_4}\sigma_{\delta \mathrm{x}_4}, \quad [101]$$

which is proportional to the uncertainty $\sigma_{\delta \mathrm{x}_4}$ in the positioning of the inverted potential.

A.3. Case study: Decline of visibility as a function of $\sigma_{\delta \mathrm{x}}$. Here we assume that all uncertainties in the positioning of the optical potentials are equal ($\sigma_{\delta \mathrm{x}_\mathrm{i}} = \sigma_{\delta \mathrm{x}}$). Then we absorb all the previously calculated blurring distances into the total blurring variance of the final interference pattern by exploiting the associative properties of convolutions

$$\sigma_{\Gamma,\mathrm{v}}^2 = \sigma_{\mathrm{v}2}^2 + \sigma_{\mathrm{v}4}^2. \quad [102]$$

Next, we replace σ_Γ by $\sigma_{\Gamma,\mathrm{v}}$ in Eq. (4) in the main text. Then we calculate the visibility of the final interference pattern of our case study (parameters in table I in the main text) as a function of $\sigma_{\delta \mathrm{x}}$. The result is shown in Fig. S7b), which indicates that for standard deviations larger than 5pm in the random shifts of optical potentials the visibility is completely lost, and for standard deviations below 1 pm no significant reduction in visibility is expected.

B. Precision errors on the pulse area during Step 2. During step 2, we propose to cancel the linear part of the optical potential (Eq. (7), main text) with an appropriate electric field pulse. This reduces the momentum kick on the particle given by the optical potential in step 2 and ensures that the particle does not leave the trapping volume. Thus, the cancellation does not have to be perfect but the residual momentum kick should be sufficiently equal for each experimental run. Here we examine how much deviation on this residual momentum kick is tolerated by the protocol. To model this, we follow a similar derivation as in section A.1, where the particle experiences a random momentum kick in each experimental run due to a shift of the potential along x. Instead, here the random momentum kick originates from a random deviation from the average pulse area $A_2 \equiv \langle A_2 \rangle + \delta A_2$. We separated the expectation value of the pulse area $\langle A_2 \rangle \equiv \omega_p^2 \tau_2$ and the random fluctuations δA_2 around the expectation value, with $\langle \delta A_2 \rangle = 0$ and standard deviation σ_{A_2}. For simplicity, we assume that the electric field pulse cancels the part proportional to $\langle A_2 \rangle$. Thus, the particle experiences a stochastic momentum kick of the form

$$\exp\left(-\mathrm{i}\frac{m}{2\hbar k}\sin(2\phi_2)\delta A_2 x\right). \qquad [103]$$

To proceed, we multiply the integrand of Eq. (4) with this momentum kick term and continue the calculation as in section A.1. Hence, the final interference pattern has to be convoluted with a Gaussian with blurring distance

$$\sigma_{\mathrm{rel}} = \frac{\sin(2\phi_2)(1+\omega_4\tau_3)}{4k\omega_4} p_{\mathrm{err}}\omega_p^2\tau_2 \exp(\omega_4\tau_4). \qquad [104]$$

in order to account for the relative precision error $p_{\mathrm{err}} \equiv \sigma_{\mathrm{A}_2}/\langle A_2 \rangle$ in the pulse area. In Fig. S7c) we show the reduction of visibility of the final interference pattern as a function of $\sigma_{\mathrm{A}_2}/\langle A_2 \rangle$ for our case study (parameters given in table I in the main text) and $\omega_p = \omega_2/\sqrt{\cos(2\phi_2)}$. We observe that precision errors up to $p_{\mathrm{err}} \sim 10^{-5}$ are well tolerated by the protocol. Note that we obtain the same requirements for the relative precision error on the pulse area of the electric field pulse during step 2.

C. Electric stray fields. Here we discuss the level of electric noise, which our protocol is able to tolerate without significantly reducing the visibility of the final interference pattern. Electric stray fields, for example coming from surface charges or charge noise on electrodes, are present during the whole experiment. This is also true for black-body radiation. Thus, in order to get an estimate on the level of tolerable noise, we calculate the power spectral density of the electric field S_E at the particle position, which would produce a localization rate comparable to black-body radiation. The single-sided spectral density of the electric field noise is given by (18)

$$S_E = 2\int_{-\infty}^{\infty} d\tau \langle \delta E_t(\tau)\delta E_t(0)\rangle e^{-\mathrm{i}\omega\tau} = \frac{4\hbar^2\Lambda_E}{e^2}, \qquad [105]$$

assuming white noise. Here, Λ_E is the localization rate due to the electric field noise, e is the charge of an electron (we assume a single charge on the particle), and $\delta E(\tau)$ are the fluctuations of the electric field at the particle position as a function of time. For the parameters of our case study (table I, main text) we obtain a localization rate for black-body radiation of $\Lambda_{\mathrm{bb}} \approx 1.9 \cdot 10^{18}\mathrm{m}^{-2}\mathrm{s}^{-1}$, (Eq. (59)). Using Λ_{bb} for Λ_E in Eq. (105) gives us $\bar{S}_E \approx 3.3 \cdot 10^{-12}\mathrm{V}^2\mathrm{m}^{-2}\mathrm{Hz}^{-1}$. Thus, we need $S_E \ll \bar{S}_E$ in order to neglect the effect of electric noise compared to the effect of black-body radiation on the final interference pattern. In Fig. 8 in (18), $S_E < 10^{-13}\mathrm{V}^2\mathrm{m}^{-2}\mathrm{Hz}^{-1}$ is measured for a distance of electrodes of 3 mm. We conclude that for our protocol electric noise can be suppressed to negligible levels with state of the art technology.

References

[S1] E Condon, Immersion of the fourier transform in a continuous group of functional transformations. *Proc. Natl. Acad. Sci.* **23**, 158–164 (1937).

[S2] H Pino, J Prat-Camps, K Sinha, BP Venkatesh, O Romero-Isart, On-chip quantum interference of a superconducting microsphere. *Quantum Sci. Technol.* **3**, 025001 (2018).

[S3] F Soto-Eguibar, P Claverie, Time evolution of the wigner function. *J. Math. Phys.* **24**, 1104–1109 (1983).

[S4] O Romero-Isart, Quantum superposition of massive objects and collapse models. *Phys. Rev. A* **84**, 052121 (2011).

[S5] R Cabrera, DI Bondar, K Jacobs, HA Rabitz, Efficient method to generate time evolution of the wigner function for open quantum systems. *Phys. Rev. A* **92**, 042122 (2015).

[S6] O Romero-Isart, Coherent inflation for large quantum superpositions of levitated microspheres. *New J. Phys.* **19**, 123029 (2017).

[S7] DE Chang, et al., Cavity opto-mechanics using an optically levitated nanosphere. *Proc. Natl. Acad. Sci.* **107**, 1005–1010 (2010).

[S8] J Bateman, S Nimmrichter, K Hornberger, H Ulbricht, Near-field interferometry of a free-falling nanoparticle from a point-like source. *Nat. comm* **5**, 4788 (2014).

[S9] S Nimmrichter, K Hammerer, P Asenbaum, H Ritsch, M Arndt, Master equation for the motion of a polarizable particle in a multimode cavity. *New J. Phys.* **12**, 083003 (2010).

[S10] DJ Wineland, WM Itano, Laser cooling of atoms. *Phys. Rev. A* **20**, 1521 (1979).

[S11] NG Van Kampen, *Stochastic processes in physics and chemistry.* (Elsevier) Vol. 1, (1992).

[S12] RJ Glauber, The quantum theory of optical coherence. *Phys. Rev.* **130**, 2529 (1963).

[S13] M Katori, H Tanemura, Zeros of airy function and relaxation process. *J. Stat. Phys.* **136**, 1177–1204 (2009).

[S14] U Delić, et al., Cooling of a levitated nanoparticle to the motional quantum ground state. *Science* **367**, 892–895 (2020).

[S15] L Magrini, et al., Real-time optimal quantum control of mechanical motion at room temperature. *Nature* **595**, 373–377 (2021).

[S16] F Tebbenjohanns, ML Mattana, M Rossi, M Frimmer, L Novotny, Quantum control of a nanoparticle optically levitated in cryogenic free space. *Nature* **595**, 378–382 (2021).

[S17] A Ranfagni, K Børkje, F Marino, F Marin, Two-dimensional quantum motion of a levitated nanosphere. *Phys. Rev. Res.* **4**, 033051 (2022).

[S18] M Brownnutt, M Kumph, P Rabl, R Blatt, Ion-trap measurements of electric-field noise near surfaces. *Rev. modern Phys.* **87**, 1419 (2015).
